# Integrating direct electrical brain stimulation with the human connectome

**DOI:** 10.1093/brain/awad402

**Published:** 2023-12-04

**Authors:** Ludovico Coletta, Paolo Avesani, Luca Zigiotto, Martina Venturini, Luciano Annicchiarico, Laura Vavassori, Sam Ng, Hugues Duffau, Silvio Sarubbo

**Affiliations:** Neuroinformatics Laboratory (NiLab), Bruno Kessler Foundation (FBK), Trento 38123, Italy; Center for Mind/Brain Sciences – CIMeC, University of Trento, Rovereto 38068, Italy; Neuroinformatics Laboratory (NiLab), Bruno Kessler Foundation (FBK), Trento 38123, Italy; Center for Mind/Brain Sciences – CIMeC, University of Trento, Rovereto 38068, Italy; Department of Neurosurgery, S. Chiara Hospital, Trento 38122, Italy; Structural and Functional Connectivity Lab Project, S. Chiara Hospital, Trento 38122, Italy; Department of Psychology, S. Chiara Hospital, Trento 38122, Italy; Department of Biotechnology and Life Sciences, Division of Neurosurgery, University of Insubria, Ospedale di Circolo e Fondazione Macchi, Varese 21100, Italy; Department of Neurosurgery, S. Chiara Hospital, Trento 38122, Italy; Structural and Functional Connectivity Lab Project, S. Chiara Hospital, Trento 38122, Italy; Center for Mind/Brain Sciences – CIMeC, University of Trento, Rovereto 38068, Italy; Department of Neurosurgery, S. Chiara Hospital, Trento 38122, Italy; Structural and Functional Connectivity Lab Project, S. Chiara Hospital, Trento 38122, Italy; Institute of Functional Genomics, University of Montpellier, CNRS, INSERM, Montpellier 34094, France; Department of Neurosurgery, Gui de Chauliac Hospital, Montpellier University Medical Center, Montpellier 34295, France; Institute of Functional Genomics, University of Montpellier, CNRS, INSERM, Montpellier 34094, France; Department of Neurosurgery, Gui de Chauliac Hospital, Montpellier University Medical Center, Montpellier 34295, France; Department of Neurosurgery, S. Chiara Hospital, Trento 38122, Italy; Structural and Functional Connectivity Lab Project, S. Chiara Hospital, Trento 38122, Italy

**Keywords:** lesion network mapping, translational neuroscience, neuromodulation targets, intracranial stimulation

## Abstract

Neurological and neurodevelopmental conditions are a major public health concern for which new therapies are urgently needed. The development of effective therapies relies on the precise mapping of the neural substrates causally involved in behaviour generation. Direct electrical stimulation (DES) performed during cognitive and neurological monitoring in awake surgery is currently considered the gold standard for the causal mapping of brain functions. However, DES is limited by the focal nature of the stimulation sites, hampering a real holistic exploration of human brain functions at the network level.

We used 4137 DES points derived from 612 glioma patients in combination with human connectome data—resting-state functional MRI, *n* = 1000 and diffusion weighted imaging, *n* = 284—to provide a multimodal description of the causal macroscale functional networks subtending 12 distinct behavioural domains. To probe the validity of our procedure, we (i) compared the network topographies of healthy and clinical populations; (ii) tested the predictive capacity of DES-derived networks; (iii) quantified the coupling between structural and functional connectivity; and (iv) built a multivariate model able to quantify single subject deviations from a normative population. Lastly, we probed the translational potential of DES-derived functional networks by testing their specificity and sensitivity in identifying critical neuromodulation targets and neural substrates associated with postoperative language deficits.

The combination of DES and human connectome data resulted in an average 29.4-fold increase in whole brain coverage compared to DES alone. DES-derived functional networks are predictive of future stimulation points (97.8% accuracy) and strongly supported by the anatomical connectivity of subcortical stimulations. We did not observe any significant topographical differences between the patients and the healthy population at both group and single subject level. Showcasing concrete clinical applications, we found that DES-derived functional networks overlap with effective neuromodulation targets across several functional domains, show a high degree of specificity when tested with the intracranial stimulation points of a different stimulation technique and can be used effectively to characterize postoperative behavioural deficits.

The integration of DES with the human connectome fundamentally advances the quality of the functional mapping provided by DES or functional imaging alone. DES-derived functional networks can reliably predict future stimulation points, have a strong correspondence with the underlying white matter and can be used for patient specific functional mapping. Possible applications range from psychiatry and neurology to neuropsychology, neurosurgery and neurorehabilitation.

## Teaser

Coletta *et al.* integrate direct electrical stimulation performed during cognitive and neurological monitoring in awake surgery with human connectome data, opening a unique window into the intrinsic organization of the human brain and providing an unprecedented holistic exploration of brain functions.

**See Elmalem *et al.* (https://doi.org/10.1093/brain/awae044) for a scientific commentary on this article**.

## Introduction

Neurological and neurodevelopmental conditions—together with mental disorders—are among the leading causes of disability, accounting for more than 9 million deaths per year.^1^ In all countries, these conditions are widespread and undertreated.^1^

New therapies, including modulation of brain regions involved in healthy brain functioning and functional recovery, are a solid and promising prospect and an urgent clinical need.^2^ Trials of both invasive and non-invasive brain stimulation techniques have targeted several brain regions, with little consensus on the optimal targets.^3,4^ In line with this uncertainty, transcranial magnetic stimulation devices cleared by the US Food and Drug Administration for the treatment of psychiatric disorders were designed to target a spatially distributed set of brain regions.^5,6^ The development of novel and effective—both invasive and non-invasive—neuromodulation therapies requires the acquisition of increasing amounts information about the cortical and subcortical neural substrates causally involved in different cognitive domains.^7^

A unique source of information that can help to address this question is direct electrical stimulation (DES), performed during cognitive and neurological monitoring in awake surgery. DES is currently considered the most reliable brain mapping strategy for identifying cortical and subcortical regions critical to functional preservation, as it has been shown to drastically improve the clinical outcome and the oncological history of patients with brain tumours.^8-11^ Crucially, DES raises the unique possibility of obtaining probabilistic functional atlases with causal information regarding the spatial configuration of several brain networks, with important scientific and clinical consequences.^8,9^ However, existing probabilistic DES-derived atlases are currently unable to provide such a mapping, as they are limited by the focal nature of the stimulation sites. Four recent studies tried to fill this gap by integrating DES with functional imaging.^12-15^ One is a single case report that used stereotactic intracerebral EEG—high temporal resolution but poor and incomplete brain coverage—to characterize the network reconfiguration induced by a manic state, while two other studies identified language and motor related subnetwork components via functional MRI without recovering the unified network subtending the corresponding behavioural domain. Lastly, Elmalem and colleagues^15^ were among the first to sketch a novel methodological framework able to integrate DES with connectome mapping. However, the authors limited their analysis to the medial wall and did not expand their analysis to include functional network properties. Taken together, this growing field of literature confirms the need for a robust procedure capable of establishing a causal link between focal electrical stimulation, behaviour, and the spatial arrangement of macro-scale functional networks of the human brain.

Recent methodological developments allowed to link lesions in different brain locations to a common neuroanatomical substrate using the human connectome,^16,17^ a detailed representation of how brain regions communicate with each other to form large-scale functional networks. In the present report, we seek to integrate the largest DES dataset ever collected in glioma patients with functional connectome mapping performed on a large normative population of 1000 healthy individuals, leveraging network hubs to build a multivariate normative model capable of quantifying single subject deviation from a reference population. In doing so, we propose to extend DES mapping toward the exploration of its network manifestation at the whole brain level and with single subject resolution, providing a viable solution to the need of causally driven brain maps for neuromodulation therapies and wide spectrum of clinical and surgical applications.

## Materials and methods

### Datasets

We used 2906 cortical and 1231 subcortical DES points derived from 592 low (WHO grade II) and 20 high grade (WHO grade III) glioma patients to provide a multimodal mapping of the networks underlying 12 functional domains. The 12 functional categories assessed during DES mapping were: verbal anomia, semantic and phonological paraphasia, non-verbal semantic association, motor and sensory responses, mentalizing, movement arrest, spatial perception, speech arrest, verbal apraxia and visual. Behavioural tasks and tumours distribution are described in the Supplementary material (see the ‘Neuropsychological testing’ section and Supplementary Table 1, respectively). Mean patient age (min–max: 38.1–40.8) was stable across functional categories, while the proportion of right handers (min–max: 81.8–94.4%) and the male-to-female ratio (min–max: 44.4–62%) showed a more pronounced variability (Supplementary Table 2). Thirty-four patients had a preoperative functional MRI scan available (20 high- and 14 low-grade glioma patients).^18^ For the purposes of the present report, cortical mapping refers to the grey matter electrically stimulated after craniotomy and before/during tumour removal, while subcortical mapping refers to the white matter bundles and/or caudate/basal ganglia that was exposed at the depth of the surgical resection cavity. A number of the DES points (1091 cortical and 681 subcortical) used in this report have already been published, together with the description of the intraoperative mapping protocol (stimulation and neuropsychological tests, the same as used in the present report) and the data annotation procedure.^8,9,19^ More details about the intraoperative mapping protocol, and pre- and post-processing data for the remaining DES points, can be found in the Supplementary material.

In addition to the 34 patients with both DES and functional imaging available, we included the preoperative resting-state functional MRI scans of 32 (25 high- and 7 low-grade) glioma patients who underwent surgery under general anaesthesia (acquisition parameters and preprocessing strategy of the combined samples are reported in the Supplementary material and a lesion loci map is presented in Supplementary Fig. 2). The fully preprocessed resting-state functional MRI dataset used for deriving the DES-driven functional networks consisted of 1000 healthy subjects and was part of the Brain Genomics Superstruct Project (GSP) dataset.^17,20^

The diffusion-weighted imaging dataset used for testing the coupling between cortical DES networks and subcortical stimulations points consisted of 284 healthy individuals (TractoInferno^21^).

### Normative network construction

**Figure 1:**
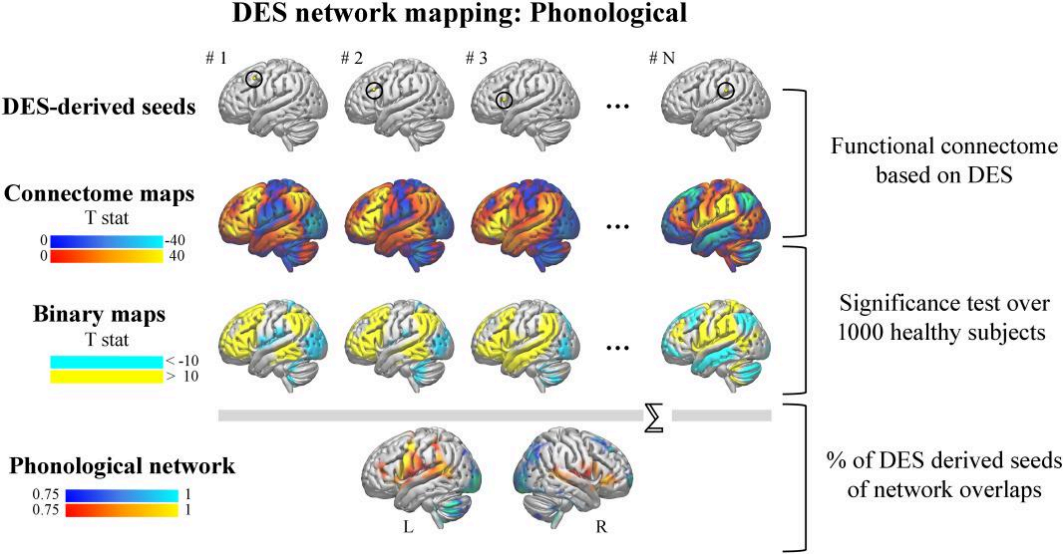
**Network construction from cortical direct electrical stimulation.** We used the direct electrical stimulation (DES) sites as seeds to derive functional maps at the group level using the functional connectome of 1000 healthy control subjects (*top two rows*; phonological DES points used for building the example). Significance testing and thresholding is achieved via a non-parametric procedure (*third row*). By aggregating binary connectivity maps for each category separately, it is possible to probe the spatial topography of causally-driven macro-scale functional networks of the human brain (*bottom row*). L = left; R = right; T stat = *t*-statistic.

**Figure 2:**
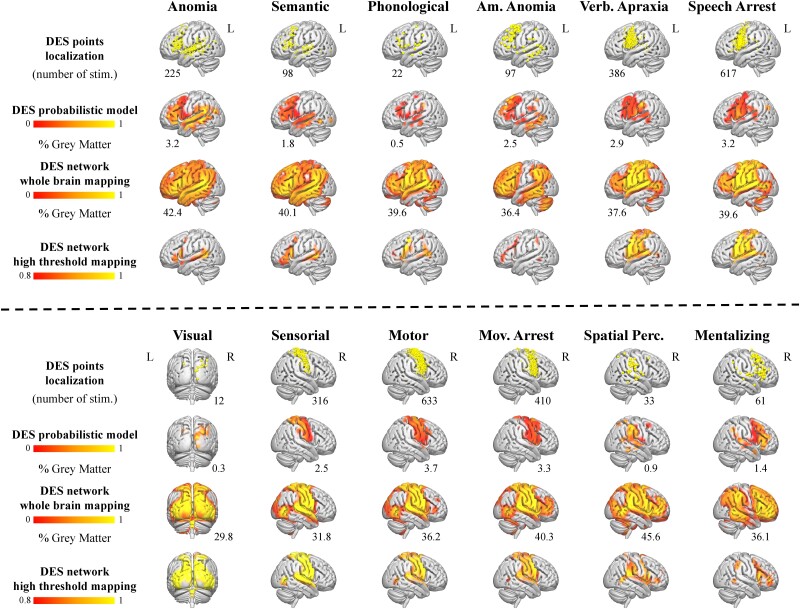
**Comparison between a traditional probabilistic direct electrical stimulation atlas of brain functions and the integration of direct electrical stimulation with connectome mapping**. We used cortical direct electrical stimulation (DES) (*top row* for each functional category) to derive a probabilistic atlas of human brain function (*second row* for each functional category). Integrating DES with connectome mapping yielded functional networks with much richer spatial topographies (*third row* for each functional category), as illustrated in the comparison of the percentage of grey matter covered with the two procedures. Setting a high threshold on the functional networks derived from the integration of DES and connectome mapping reveals function specific network organizations (*bottom row* for each functional category). Am. Anomia = amodal anomia; L = left; Mov. Arrest = movement arrest; R = right; Spatial Perc. = spatial perception; stim. = stimulations; Verb. Apraxia = verbal apraxia.

**Figure 3:**
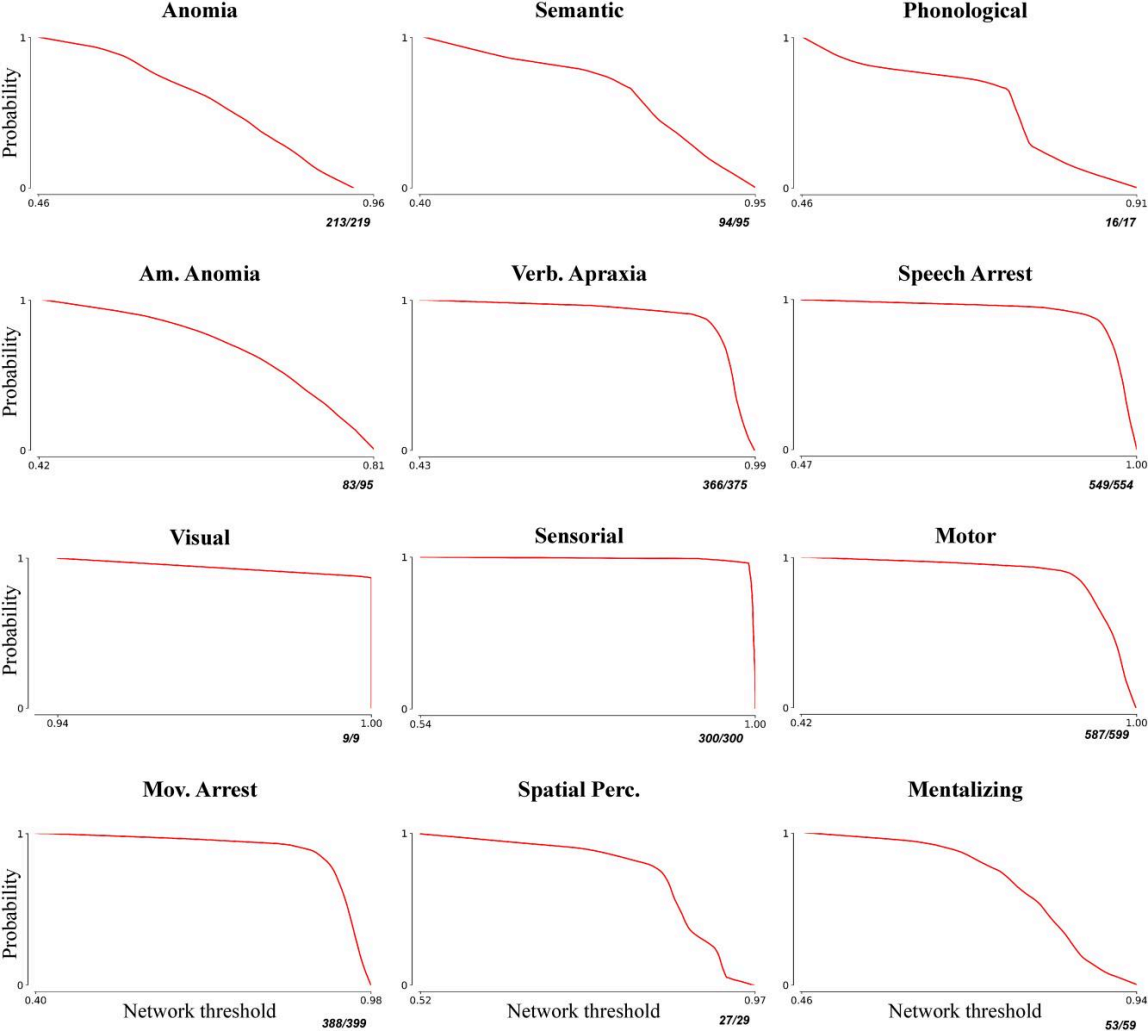
**Exceedance curves and accuracy values for the correctly classified direct electrical stimulation sites.** For each category, we repeated the network construction procedure by iteratively leaving out one direct electrical stimulation (DES) point. The left-out point not included in the network construction phase was used to contrast the DES positive against the DES negative network, reasoning that if functional connectivity is a proxy for a shared function, then the left-out point not included in the DES-derived networks should fall in a brain regions characterized by high positive functional connectivity and hence higher frequency for the DES positive network than for the negative network. Here, we described the frequency distribution in the DES positive network for the correctly classified left-out points, plotted as the probability of finding an unseen stimulation point (*y*-axis) above a certain network threshold (i.e. frequency) value (*x*-axis). The results suggest a clear distinction between functional categories centred on primary sensory regions and cognitively demanding functional categories relying on widely distributed functional networks, while the overall high accuracy values confirm the predictive capacity of our functional mapping. Am. Anomia = amodal anomia; Mov. Arrest = movement arrest; Spatial Perc. = spatial perception; Verb. Apraxia = verbal apraxia.

We used lesion network mapping (LNM) and the GSP dataset to derive functional networks from the set of DES cortical points.^16,17^ By using the lesions’ locations associated with a certain syndrome to derive group representative functional connectivity maps obtained from a large cohort of healthy subjects, LNM leverages the spatial heterogeneity in lesion location to extract a common neural substrate associated with that syndrome. Similarly, we treated each cortical DES point as a transient lesion, using it as a seed to derive functional maps (Fig. 1). For each functional category, we obtained a single brain map expressing at each voxel location the percentage of DES-derived seeds with statistically significant functional connectivity separately for the positive (functionally correlated, Fig. 2) and negative (functionally anti-correlated) networks by using a non-parametric procedure [Randomise, as implemented in the FMRIB Software Library (FSL)^22^]. To quantify the gain in brain coverage provided by the combination of DES and functional imaging, we compared the spatial extension of DES-derived networks in the grey matter with the coverage provided by a probabilistic model based on DES points alone (Fig. 3).^8^ More details about LNM implementation can be found in the Supplementary material.

### Validation

We implemented three different analyses to validate our approach. First, we computed the degree of similarity (via Pearson correlation, corrected for spatial autocorrelation)^23^ between the group average DES-driven functional maps of the glioma patients and the group average DES-driven functional maps of the healthy population, leveraging the DES points and functional imaging scans of 34 patients with preoperative functional imaging available. We restricted the comparison to functional categories of clinical interest—sensory-motor functions and language—and with at least 10 stimulation points available. Following these criteria, the functional categories anomia (*n* = 22), speech arrest (*n* = 31), sensorial (*n* = 12) and motor (*n* = 30) were retained for further analysis. Further methodological details, together with an additional analysis, where we further split the glioma patients into high- and low-grade, are reported in the Supplementary material.

Second, we tested whether DES-derived networks are predictive of future stimulation points via cross validation (Fig. 3). For each category, we repeated the network construction procedure by iteratively leaving one DES point out of the network building procedure, using it as a benchmark to test the predictive power of both the DES positive and negative derived functional networks estimated using all the other DES stimulations. The left-out point not included in the network construction phase was used to contrast the DES positive against the DES negative network, reasoning that if functional connectivity is a proxy for a shared function, then the left-out point not included in the DES-derived networks should fall in a brain region characterized by high positive functional connectivity and hence higher frequency for the DES positive network than for the negative network. Concretely, the left-out point was used to compute the mean concordance rate for the positive and negative network separately, and the DES network was classified as predictive if the average concordance rate for the positive network was greater than the average concordance rate of the negative network. In the Supplementary material, we present additional analyses in which we tested the specificity and robustness of DES-derived networks, as well as the impact of repeated DES measurements within our patients’ cohort in the network construction phase (Supplementary Figs 3 and 4 and ‘Validation analyses’ section, ‘Repeated DES measurements of the same patient’ subsection, respectively).

**Figure 4:**
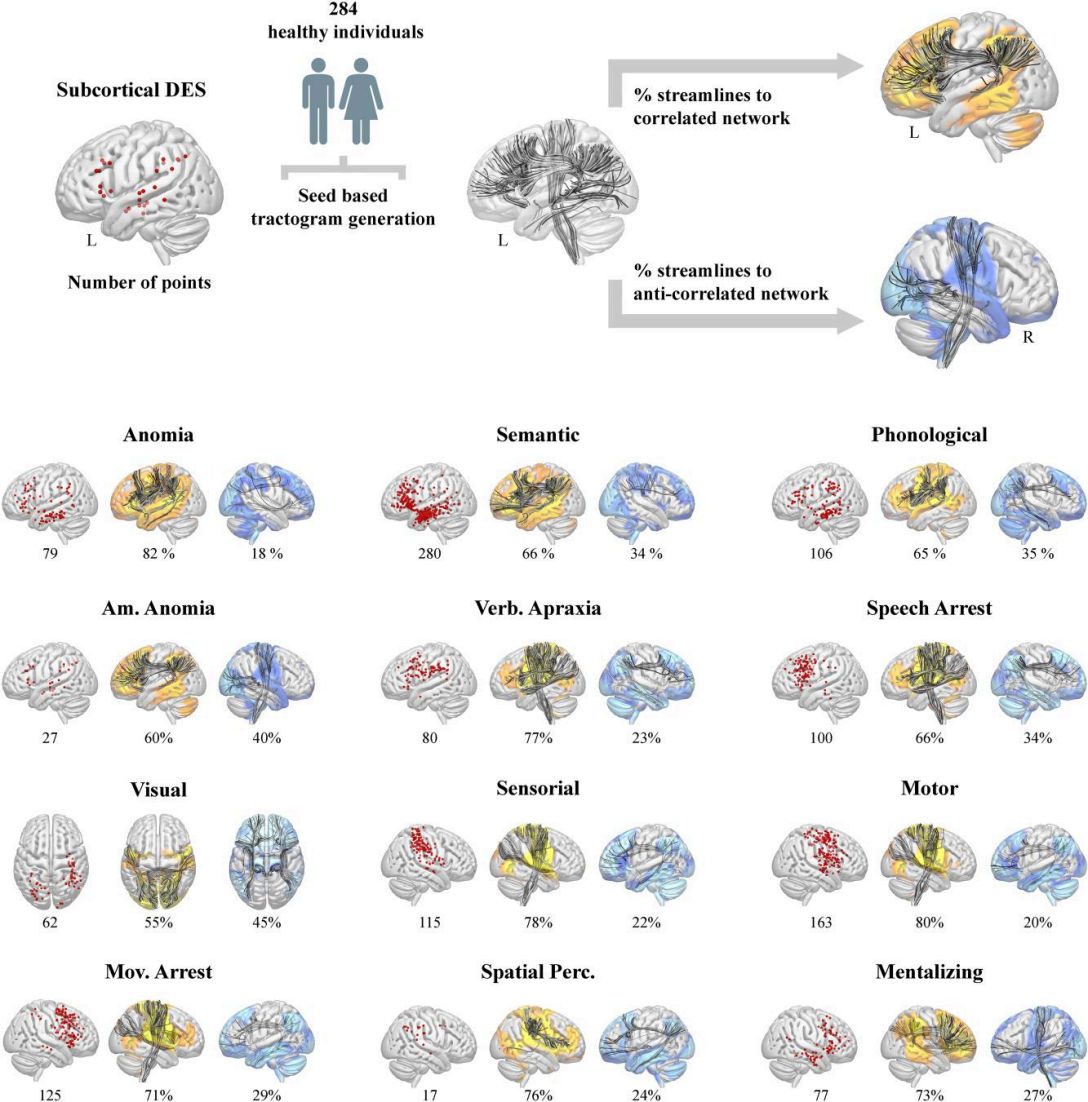
**Integration of subcortical direct electrical stimulation with functional mapping.** Number of subcortical direct electrical stimulation (DES, red circles for each functional category) and schematic representation of the difference in structural connectivity density between subcortical DES and positive/negative DES-derived networks (*middle* and *right* column for each functional category, respectively). We also reported the percentage of reconstructed streamlines connecting the subcortical DES to the corresponding DES positive and negative functional networks, averaged across stimulation sites and subjects. Am. Anomia = amodal anomia; L = left; Mov. Arrest = movement arrest; R = right; Spatial Perc. = spatial perception; Verb. Apraxia = verbal apraxia.

Last, we used the TractoInferno dataset^21^ to test whether DES-derived cortical networks are supported by the structural connectivity of the subcortical stimulations. Specifically, we first generated for each of the 284 subjects a functional category specific tractogram (i.e. 284 × 12 tractograms generated via constrained spherical deconvolution deterministic tractography). In a second step, we extracted the percentage of streamlines connecting each subcortical DES point to the corresponding DES positive and negative functional network, averaging across subjects to obtain the numbers reported in Fig. 4 (see the Supplementary material for a detailed description).

### Single subject analysis

**Figure 5:**
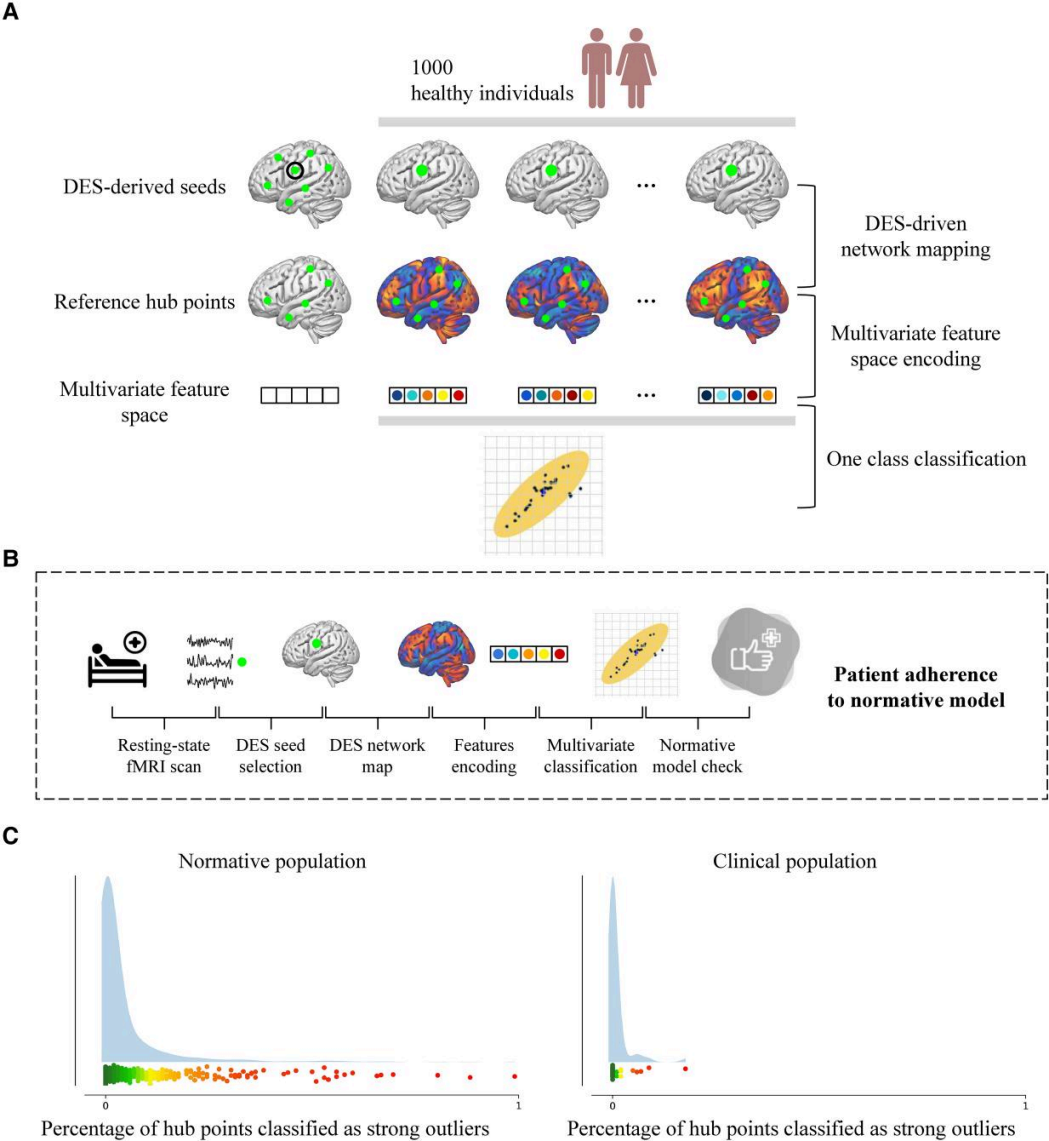
**Illustration of the pipeline for single subject analysis and comparison between a clinical and reference population.** Leveraging direct electrical stimulation (DES) sites falling within core functional regions, it is possible to build a multivariate model able to distinguish between in- and outliers (**A** and **B**), offering a tool to examine how the functional architecture at the single subject level relates to the functional architecture of a normative model (**C**). fMRI = functional MRI.

To highlight the potential deployment of DES-derived networks in clinical practice, we implemented a multivariate outlier detection analysis able to quantify the adherence of the single subject functional organization to the mapping obtained at the group level (Fig. 5A and B and Supplementary material). As for the validation analysis, we initially restricted the computation to the same subset of 95 cortical DES points derived from the 34 patients with both DES and preoperative functional imaging available (see the ‘Validation’ section for more details). Next, we repeated the procedure to build a classifier specific for language and derived from stimulation points that fell—for each category independently—within hub territories of anomia, semantic and phonological. In this latter analysis, we also included the functional scans of the glioma patients that underwent surgery without DES mapping (final *n* = 66). The hub threshold was set at the 90th percentile of each functional category as per cross validation procedure (see the ‘Validation’ section). Cross validation (leave one out) was also performed on the healthy population to build a reference distribution. Hub points with a classification score above the median of the negative values of the reference population were labelled as strong outliers. In addition, we employed a non-parametric procedure to compare the distribution of the two samples. Of the 1000 healthy control subjects, we randomly selected 66 participants, repeating the procedure 100 000 times (sampling with replacement). In each iteration, we performed a two sample *t*-test, comparing the randomly extracted 66 healthy subjects with the 66 patients and extracting the effect size (as per Cohen’s *d*) as the measure of interest (Supplementary material and Supplementary Fig. 5).

### Clinical validation

**Figure 6:**
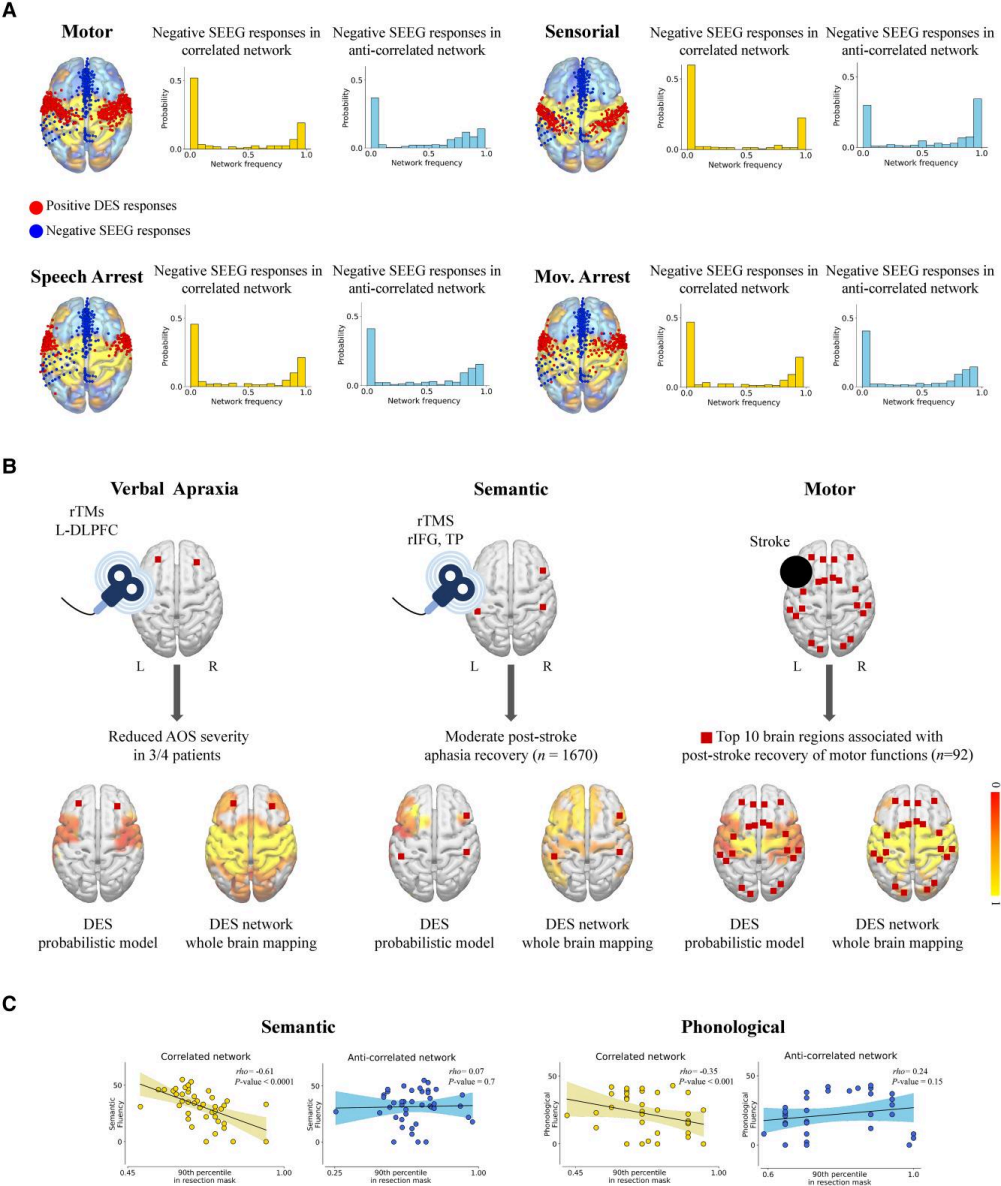
**Clinical validation of direct electrical stimulation-derived functional networks.** (**A**) For each functional domain of interest, we superimposed the direct electrical stimulations (DESs) resulting in no behavioural disruption (blue, 241 stereoelectroencephalography (SEEG) stimulations obtained) and DESs resulting in behavioural disruption (red, see the ‘Materials and methods’ section) on top of the corresponding bipartite functional networks. As shown in the histograms, our analysis revealed that SEEG negative responses robustly co-localize with highly peripheral regions of the DES positive functional networks, highlighting their specificity. (**B**) Brain regions subtending partial recovery from apraxia of speech, stroke induced aphasia and the post-stroke recovery of motor functions spatially overlap with the corresponding DES-derived networks—but not with functional atlases based on DES alone (*right* to *left*). (**C**) DES positive but not DES negative networks contribute to the behavioural deficits observed in the language domain 1 week after surgery, as shown by the correlation between the importance of the surgically removed DES positive/negative networks of phonological and semantic with the corresponding behavioural score for phonological and semantic fluency. AOS = apraxia of speech; DLPFC = dorsolateral prefrontal cortex; IFG = inferior frontal gyrus; L = left; R = right; rTMS = repetitive transcranial magnetic stimulation; TP = temporoparietal region.

To corroborate the predictive analysis described in the ‘Validation’ section, we performed a mixed literature overview/empirical analysis aimed at probing whether DES-derived networks can be used in combination with both invasive and non-invasive stimulation techniques or used to characterize the neural substrates of psychiatric disorders (Figs 6A and B, Supplementary material and Supplementary Fig. 6, respectively). Lastly, we leveraged DES-derived networks to investigate the transient post-surgical behavioural deficits (Fig. 6C).

Owing to the absence of negative DES responses (i.e. stimulations resulting in no behaviour disruption) in our dataset, we tested the specificity of DES-derived functional networks against the data of Elmalem and colleagues (Fig. 6A).^15^ In this study, the authors used 477 DES stimulations obtained via stereoelectroencephalography (SEEG) from 37 patients in combination with normative data to investigate the connectional properties of the medial wall, recording the Montreal Neurological Institute (MNI) location of both DES positive (i.e. stimulations resulting in behaviour disruption) and DES negative (i.e. stimulations resulting in no behaviour disruption) responses. We focused on the latter ones, testing the DES negative responses against our DES-derived functional networks of motor, movement arrest, speech arrest and sensorial, i.e. the same four behavioural categories analysed by Elmalem *et al*.^15^ After data inspection, we removed one duplicated stimulation point and one point due to no overlap with our grey matter mask, resulting in *n* = 241 stimulation points used for the analysis. We compared the distribution of DES negative responses in the DES positive and DES negative networks via Wilcoxon signed-rank test (as implemented in Scipy v1.7.3).^24^

To probe whether DES-derived networks can be used to identify novel neuromodulation targets, we performed a qualitative literature overview. Two out of three identified studies investigated the effects of transcranial magnetic stimulation for the recovery of language functions in apraxia of speech disorder (Fig. 6B, left panel)^25^ and after a stroke (Fig. 6B, middle panel),^26^ while the third identified study focused on the distinction between patients who exhibited natural recovery of motor function and patients who did not exhibit a recovery of motor functions after a stroke^2^ (Fig. 6B, right panel). The set of targeted/identified regions was quite diverse; left dorsolateral prefrontal cortex for apraxia of speech; right inferior frontal gyrus, right and left temporoparietal regions for post-stroke recovery of language functions; and a wide distributed network for motor recovery. To visually probe the spatial correspondence between our mapping procedure and the results reported in the literature, we derived regions of interest from a set of different atlases. We extracted centre of gravity coordinates for the dorsolateral prefrontal cortices from the Brainnetome atlas,^27^ recently described as optimally suited for localizing transcranial magnetic stimulation targets in individual anatomical space.^28^ Right and left inferior frontal regions were extracted from the AAL atlas v3,^29^ while the temporoparietal region was defined directly in MNI space by hand by the first author (L.C.). The centres of gravity of the top 10 brain regions associated with the recovery of motor functions were extracted directly from the atlas^30^ used in the corresponding study^2^ by running Freesurfer’s recon-all pipeline in MNI space.^31^

To disentangle the contribution of DES positive and DES negative networks to postoperative neurosurgical deficits in the language domain, we retrospectively reanalysed the 42 patients described by Zigiotto *et al*.^32^ For each patient in the dataset, we extracted the portion of his/her surgical cavity belonging to either the DES positive or DES negative functional network (on the basis of the bipartite networks, for phonological and semantic separately). For each of these two masks, we computed the 90th percentile of the corresponding DES network and we correlated the two values—one for the positive and one for the negative DES network—with the phonological and semantic fluency scores obtained 1 week after surgery.

### Ethical statement

The study was approved by the ethical committee of the APSS (Project: NeuSurPlan). All the data were collected according to the procedure approved by the Internal Review Boards and in agreement with The Code of Ethics of the World Medical Association (Declaration of Helsinki).

## Results

### From direct electrical stimulation points to macroscale functional networks

We used 2906 cortical and 1231 subcortical DES points derived from 592 low (WHO grade II) and 20 high grade (WHO grade III) glioma patients in combination with LNM (Fig. 1)^33^ to provide multimodal mapping of the causal functional networks underlying 12 distinct functional domains/categories (Fig. 2, ‘Materials and methods’ section and Supplementary material). By using the lesions’ locations associated with a certain syndrome as seeds to derive group representative functional maps obtained from a large cohort of healthy subjects, LNM is able to leverage the spatial heterogeneity in lesions location to extract a common neural substrate associated with that syndrome.^16^ Similarly, we treated each cortical stimulation point as a transient lesion and used it as a seed for performing a seed based analysis^34^ in a normative connectome derived from 1000 healthy individuals (GSP dataset).^17,20^ After binarization via a non-parametric procedure,^22^ we aggregated DES driven functional maps to obtain the macroscale functional network subtending the functional category of interest, separately for the positive (i.e. functionally correlated; Fig. 2) and negative (i.e. functionally anti-correlated) networks.

Concerning the spatial distribution of DES points, we found language related functional categories predominantly left lateralized, while mentalizing and spatial perception stimulation points were found more often in the right hemisphere (Supplementary Table 2). Sensory-motor and motor planning networks (i.e. verbal apraxia, speech arrest and negative motor responses) showed an expected bi-hemispherical representation. The functional distribution of all the networks explored were concordant with the literature, including bi-hemispherical support previously described for language elaboration.^8,35,36^ The combination of DES and functional imaging resulted in an average 29.4-fold increase in whole brain grey matter coverage compared to the state of the art probabilistic network building procedure provided by DES points alone,^8^ with this quantity varying between 9.84 and 92.93, depending on the number of DES points of the functional category considered (Fig. 2). Of note, DES-derived networks are presented at two granularity levels—i.e. two different frequency thresholds—to show the existence of widespread and overlapping functional systems that nonetheless possess a high degree of specialization.

### Probing the validity of direct electrical stimulation-derived networks at the group level

By providing functional connectivity maps at the voxel level, seed based analysis allows regionally unconstrained mapping of whole brain connectivity and, combined with seeds derived from DES, it allows causal mapping of the spatial topography of putative large-scale functional networks subtending each tested functional category. To probe the soundness of our mapping procedure, we implemented a series of additional analyses. First, we computed the degree of similarity (via Pearson correlation, corrected for spatial autocorrelation)^23^ between the group average DES driven functional maps of the glioma patients and the group average DES driven functional maps of the healthy population, leveraging the DES points and functional imaging scans of 34 patients with preoperative functional imaging available. Although modest in size, this dataset provides the unique opportunity to test the correspondence between DES and connectome mapping within the same individuals. Within this subset of patients, we restricted the comparison to functional categories of clinical interest—sensory-motor functions and language—and with at least 10 stimulation points available. Following these criteria, the functional categories anomia (*n* = 22), speech arrest (*n* = 31), sensorial (*n* = 12) and motor (*n* = 30) were retained for further analysis. We found that the group average DES driven functional maps of the glioma patients and group average DES driven functional maps of the healthy population did not differ for the subset of 95 DES points tested in the validation analysis, even after accounting for spatial autocorrelation (average Pearson *r* = 0.85, corrected *P*-values < 0.0001, 1000 surrogates per single DES point). Further methodological details, together with an additional analysis where we further split the glioma patients in high- and low-grade are reported in the ‘Materials and methods’ section and in the Supplementary material.

Second, we tested whether DES-derived networks are predictive of future stimulation points via cross validation (see the ‘Materials and methods’ section for further details). Indeed, our analysis revealed that DES-positive networks are predictive of future stimulation points (97.8% accuracy). The median concordance rate for the left-out points—computed across functional categories—was much higher for the DES positive networks than for the DES negative networks (0.95 and 0.02, respectively, 25th and 75th percentile = 0.89 and 0.98 for DES positive and 0.01 and 0.06 for the DES negative networks, respectively). In Fig. 3, we described the frequency distribution in the DES positive network for the correctly classified left-out points, plotted as the probability of finding an unseen stimulation point (*y*-axis) above a certain network threshold (i.e. frequency) value (*x*-axis), a so-called exceedance curve. Our analysis highlighted a clear difference between functional categories centred on primary sensory regions and cognitively demanding functional categories relying on widely distributed functional networks.

Last, we used the TractoInferno^21^ dataset to test whether DES-derived cortical networks are supported by the structural connectivity of the subcortical stimulations (Fig. 4, top row). Probing the structural connectivity of subcortical DES points unveiled a tight coupling between structure and function, as subcortical DES points for a given functional category were found to preferentially connect to the corresponding DES positive network compared with the corresponding DES negative network (Fig. 4).

### Probing the validity of direct electrical stimulation-derived networks at the single subject level

Although the integration of DES and connectome mapping yielded a robust and reliable mapping at the group level, it is not able to capture single subject deviations from a normative population, resulting unable to satisfy the critical need of a subject (or patient) specific functional mapping.^37^ To fill this critical gap, we implemented a multivariate outlier detection analysis able to quantify the adherence of the single subject functional organization to the mapping obtained at the group level (Fig. 5A and B). As for the validation analysis, we initially restricted the computation to the same subset of 95 cortical DES points derived from the 34 patients with both DES and preoperative functional imaging available (see the ‘Validation’ section for more details). Next, we repeated the procedure to build a classifier specific for language and derived from stimulation points that fell—for each category independently—within the networks hub territories of anomia, semantic and phonological. In this latter analysis we also included the functional scans of the glioma patients that underwent surgery without DES mapping (final *n* = 66, see the ‘Materials and methods’ section and Supplementary material). The distribution of strongly deviant language hubs showed a prominent right skewed distribution in both clinical and normative populations (Fig. 5C, bottom row), with the vast majority of individuals with no or very few strongly deviant hub points and a small percentage of both patients and healthy subjects characterized by a strongly deviant network architecture. Our non-parametric analysis revealed that the two populations were characterized by Cohen’s *d* ≤ 0.52 for 80% of the time, thus indicating small/moderate differences in the functional architecture of the hub points (Supplementary Fig. 5). In line with this result, none of the 95 DES points belonging to the subset of 34 patients with functional imaging available was classified as an outlier.

### Clinical validation

After testing the methodological soundness of our mapping procedure, we performed a series of mixed literature overview/empirical analysis aimed at highlighting potentially useful clinical applications.

We first tried to complement the predictive capacity of DES-derived networks by testing their specificity against 241 DES stimulations obtained via SEEG and not resulting in behaviour disruption. Importantly, the comparison with a different stimulation technique represents an important testing ground, as it probes the generalizability of our mapping procedure. As shown in the histograms presented in Fig. 6A, our analysis revealed that SEEG negative responses robustly co-localize with highly peripheral regions of DES positive functional networks. We also found a residual fraction of SEEG negative responses in prominent regions of the DES positive functional networks, likely driven by the overlap between the negative SEEG responses of Elmalem *et al*.^15^ and the positive DES responses from our intraoperative testing, as illustrated in the 3D renderings of Fig. 6A contrasting DES positive (red) and SEEG negative points (blue) on the corresponding bipartite functional networks. Here we argue that this overlap is also partly obscuring another potential result, namely the higher prevalence of negative SEEG points in the DES negative network, as the comparison with the DES positive network is statistically significant for sensorial only (median in positive network = 0, median in negative network = 0.76, *t* = 12010, *P* < 0.05 as per Wilcoxon rank sum test).

Second, we performed a qualitative literature overview to probe whether DES-derived networks can be used to identify novel neuromodulation targets. We found that brain regions subtending partial recovery from apraxia of speech,^25^ stroke induced aphasia^26^ and the post stroke recovery of motor functions^2^ spatially overlap with the corresponding DES-derived networks—but not (or only minimally) with functional atlases based on DES alone (left, middle and right panels of Fig. 6B, respectively), highlighting the potential high degree of sensitivity of our mapping procedure.

Third, we investigated a potential role of DES-derived networks in characterizing the transient postoperative deficits in the language domain by correlating the importance of the surgically removed DES positive/negative networks of phonological and semantic with the corresponding behavioural postoperative score for phonological and semantic fluency. As shown in Fig. 6C, we found a strong negative correlation between the importance of the resected DES positive network for semantic and the semantic fluency score (the lower the score, the higher the impairment), while no relationship was found when we tested the DES negative network (Fig. 6C, left panel; Spearman’s *ρ* = −0.61, *P* < 0.0001 and Spearman’s *ρ* = 0.07, *P* = 0.7 for the DES positive and DES negative, respectively). Similarly, we observed a negative correlation between the importance of the resected DES positive network for phonological and the phonological fluency score and no correlation for DES negative network (Spearman’s *ρ* = −0.35, *P* < 0.001 and Spearman’s *ρ* = 0.07, *P* = 0.65, respectively). Of note, for the DES phonological network, we found six patients with a perfect bipartite surgical cavity (e.g. completely belonging to the DES positive or DES negative network). Removing those patients from the analysis did not significantly affect the results (Fig. 6C, right panel; Spearman’s *ρ* = −0.35, *P* < 0.001 and Spearman’s *ρ* = 0.24, *P* = 0.15 for the DES positive and DES negative network, respectively). Consequently, the scatter plots in Fig. 6C were generated without the six patients.

Lastly and as reported more in depth in the Supplementary material, we found a moderate positive correlation between a summary metric reflecting the intensity of local spontaneous activity in the dorsolateral prefrontal cortices—key regions of the causal network subtending mentalizing ability—and impairment in social behaviour in autism spectrum disorder patients (Spearman’s *ρ* = 0.21 and 0.22, for the left and right dorsolateral prefrontal cortices, respectively, *P* < 0.02).

Taken together, our preliminary results highlight the potential neuroscientific and clinical potential of our mapping procedure, as highlighted by the high degree of specificity and sensitivity in capturing both invasive and non-invasive neuromodulation targets.

## Discussion

In the present report, we propose a novel framework able to integrate DES and functional imaging, providing an unprecedented description of the networks associated with the stimulation sites of 12 distinct functional domains. Our results showed that the integration of DES and functional imaging results in a massive increase in brain coverage compared to the spatial distribution of the stimulation points only. Critically, we found that DES-derived networks are predictive of future stimulation points, confirming the causal nature of LNM applied to DES. Furthermore, we also assessed the validity of transferring DES points obtained from a set of patients to a healthy population by contrasting a large dataset of healthy individuals with a valuable dataset of patients where both DES mapping and functional imaging were available. Corroborating the soundness of our mapping, we found robust above-chance similarities in the averaged network representations of the normative population and the patients. We also implemented an additional proof-of-concept analysis in which we probed the coupling between structure and function by linking the subcortical stimulation points to the DES-derived networks. Underscoring the validity of our procedure, we found strong structural connectivity between subcortical stimulations points and DES positive functional networks. Lastly and to highlight the potential deployment of DES-derived networks in clinical practice, we leveraged the subset of individuals with both preoperative functional imaging data and DES points available to implement a multivariate normative model capable of quantifying single subject deviation from a reference population. Our analysis suggested that the functional architecture of this subset of patients—tested at the single subject level—is highly concordant with the normative population, a result that was further replicated by increasing the size of the clinical population and by using a language specific classifier.

The integration of DES and connectome mapping offers a unique window into the intrinsic organization of the brain, providing a real holistic exploration of human brain functions with important clinical and scientific consequences and advancing our understanding of the fundamental architectural principles of the human brain in two main directions. First, the integration of DES and connectome mapping yields mechanistic insights at the network level that cannot be achieved with DES or MR imaging alone, as highlighted by the prediction power of the DES-derived networks and the increase in brain coverage compared to the spatial distribution of the stimulation points only. Of note, the additional inclusion of structural connectivity further corroborated the specificity and biological plausibility of our results, as DES positive networks seemed to be characterized by a strong structural scaffold.

The second notable contribution of our work lies in the opportunity of quantifying the adherence of the single subject functional organization to the causal mapping obtained at the group level. While our results suggest a small/moderate difference between patients and normative population in the functional architecture of hub points, thus strongly corroborating the soundness of our mapping, the informative value of the single subject analysis is not limited to the validation of a computational technique. Here, we propose the integration of DES and functional imaging, in combination with the single subject pipeline, as new disruptive tools able to delineate both invasive and non-invasive stimulation targets, breaking possible confirmation biases and expanding our exploration of the human brain. To further corroborate this latter point, in our clinical validation analysis we found that DES-derived functional networks overlap with effective neuromodulation targets across several functional domains, show a high degree of specificity when tested with the intracranial stimulation points of a different stimulation techniques and can be used effectively to characterize both postoperative behavioural deficits and neurodevelopmental disorders.

Motivated by preliminary results showing that ablation of important DES positive but not DES negative network regions is strongly related to the behavioural deficits observed 1 week after the surgery, we argue that DES-derived networks can also play a critical role in surgery planning. For example, DES positive networks could be used in combination with the functional scan of a new patient to probe the single subject adherence to the maps obtained at the group level, identifying possibly deviant brain regions. In doing so, it would be possible to spare function specific network hubs from being resected when awake surgery and DES mapping are not possible, avoiding irreparable functional damage. Of note, the scope of possible applications is not limited to neurosurgery and neuromodulation but it is potentially much broader. We envisage possible application of DES-derived networks as novel tools to gain new insights on cognitive syndromes and specific cognitive impairment based on cortical-subcortical network disruption, or to explore and define brain plasticity mechanisms in pathological and healthy subjects.

While our multimodal procedure delineates a robust framework able to provide a causal mapping of brain networks in the human brain, several limitations also exist. First, LNM allows causal inferences at the network level only and not at the level of single brain regions.^16^ This is also true for hub regions, which, despite their critical role in defining the network, may or may not be critical for producing a certain behaviour. Second, it is not possible to combine networks in a causal way, as this would require stimulation points for all the possible network combinations, which is unfeasible given the obvious intraoperative time limitations. Third, the sample size of the clinical population was rather modest in size, limiting the explanatory power of the network topographies comparison between patients and the healthy population and both single subject clinical validation analyses. A larger clinical sample may be used to establish a link between deviant functional architecture and cognitive and/or genetic factors and to replicate the preliminary results of our clinical validation. Last, DES mapping is intrinsically associated with selection and sampling biases and limited brain coverage. Stimulations are driven by the tumour’s location, some brain areas are difficult to reach (e.g. the cerebellum) and the acquisition of negative stimulation data is often not implemented, as it requires reviewing—if available—every intraoperative video recordings multiple times. A more balanced acquisition of positive/negative responses and a more comprehensive spatial coverage of the DES points may be used to redefine the networks, leading to more accurate functional maps.

In summary, the integration of DES with the human connectome fundamentally advances the quality of the functional mapping provided by either DES or functional imaging alone. DES-derived networks can reliably predict future stimulation points, have a strong correspondence with the underlying white matter and can be used for single subject specific functional mapping, with important consequences for neuromodulation therapies and preoperative neurosurgical planning.

## Supplementary Material

awad402_Supplementary_Data

## Data Availability

Cortical and subcortical DES coordinates in MNI space, anonymized patients’ demographic data, aggregated functional maps for the 12 functional categories and the scripts used in this manuscript are available on Zenodo (https://zenodo.org/records/10439149) and Github (https://github.com/FBK-NILab/intraopmap), respectively.
