# Supplementary material for: Integrating direct electrical brain stimulation with the human connectome
Source: Brain. 2023 Dec 4;147(3):1100–11. doi: 10.1093/brain/awad402 (PMC10907080; doi:10.1093/brain/awad402)
Supplement: awad402_Supplementary_Data [file awad402_supplementary_data.pdf]

Supplementary Material

Direct Electrical Stimulation (DES) intraoperative mapping

We followed the principle of functionally guided tumour removal.[1,2] Practically, the principle of functionally guided tumour removal entails that the tumour removal is initiated only after completion of the direct electrical stimulation (DES) cortical mapping, and administered until positive DES subcortical responses are obtained. Of note, every stimulation site is repeated three times during the surgery, and the stimulation is categorised as “functional” – and annotated with a tag placed on the brain’ surface – if it induces a behavioural disturbance at least 2/3 times in a nonconsecutive manner. Stimulation sites that do not reach this level of reproducibility are categorised as “non-functional”. In doing so, the technique avoids possible cognitive deficits even in territories not classically and properly considered functional, such as the frontal pole and the mesial surfaces.

Neuropsychological testing

Concerning the description of the behavioural tasks, we are reporting here the details of the twelve functional categories.[1]

Motor & Movement Arrest

Motor functions were monitored in two ways: (1) overt muscle twitch noted during cortical or subcortical stimulation (positive motor responses) and (2) alteration of a continuous complex motor task (simultaneous hand, arm, and forearm flexion-extension contralateral to the lesion side) during stimulation, specifically acceleration, deceleration, or halting of movement without loss of muscle tone and without alteration of consciousness (negative motor responses).

Sensorial

Sensory responses were reported verbally by the patient during cortical and subcortical mapping, in particular dysesthesias of the contralateral face, arm, hand, or leg during stimulation.

Speech Arrest

Spontaneous language production was monitored by a counting task (series from 0 to 10) and repetition test, with positive functional responses being involuntary cessation of counting or inability to repeat, respectively, during stimulation.

Phonological, Semantic, Anomia

Phonological and semantic aspects of language processing (i.e. semantic and phonological paraphasias or pure anomia, not related to motor/praxis/visual disturbances) were assessed with picture naming test [denomination object 80 (DO 80)] Examples of positive functional responses included semantic paraphasias (e.g. patient says “dog” instead of cat during presentation of a picture of a cat), phonemic paraphasias (e.g. patient says “kite” instead of cat after presentation of a picture of a cat), and anomias (e.g. patient states “this is a ..” but cannot come up with “cat” during presentation of a cat picture).

Amodal Anomia

Non-verbal semantic disorders (namely, asemantism) were identified with the pyramid-palm-tree test (PPTT). For the PPTT, a picture is shown, and the patient asked to select the one picture (from a choice of two) that goes with the original picture (e.g. the choice between a figure of a “palm” and a figure of a “pine tree” which match with the main picture of a “pyramid”).

Visual

Visual functions were monitored as follows: a computer screen was divided in four virtual quadrants, separated by dotted lines, with a cross in the centre. Two different pictures (from DO 80) were presented together, the former in the visual quadrant affected by the tumour, i.e. the one to preserve during surgery, the latter in the opposite visual quadrant. The patients were asked to name both pictures, fixing the centre of the screen. The neuropsychologist collected the functional response errors (blurred vision, shadows, agnosia) and eventual spontaneous saccades. Moreover, a blank screen separated in quadrants by dotted lines was also used to monitor positive visual responses elicited by DES, such as flashes and phosphenes.

Verbal Apraxia

Impairments in verbal articulation were monitored by the neuropsychologist during patients' spontaneous speech as well as during a counting task (from 0 to 10) and repetition of series (days of the week, months of the year).

Spatial Perception

Spatial cognition was assessed with a line bisection task, in which the patient is presented with a standard length line and asked to mark the midpoint with a pen.

Mentalizing

Functional responses related to mentalizing (i.e. understanding, and consequently predicting, mental and psychological states and behaviours) were assessed using a variant of the classical Reading the Mind in the Eyes Test, in which the patient is presented with a set of eyes and then asked to choose (from a list of two) the emotional state.

Importantly, during these tasks, both the patient and neuropsychologist/speech therapist were blinded to the timing of DES performed by the surgeon**.**

DES points annotation

The stimulation locations are annotated with respect to the patient specific post-operative MRI scan. Specifically, this technique is based on the localization of the DES point on a 3 plane reconstruction, comparing the gyral and vascular anatomy with the intra-operative digital high-resolution pictures coming from the detailed surgical reports. The procedure is carried out by a neurosurgeon and expert anatomist, and revised by a second neurosurgeon. Of note, the procedure is largely adopted by the community [1–4] and characterised by a high degree of reliability at both cortical and subcortical level.[2,5] Roux and colleagues reported that the mean ± SD of the absolute difference between both operators was 0.08 mm ± 3.36 for the x-axis, 1.27 mm ± 5.12 for the y-axis, and 0.87 mm ± 6.1 for the z-axis, respectively.

DES points standardisation

STEP: Data Preprocessing

As mentioned in the *Methods* section (see main text), 1'091 cortical and 681 cortical DES points used in this report have already been published,[6] together with the description of the

intraoperative mapping protocol (stimulation and neuropsychological tests, same used in the present report) and the data annotation procedure. Here, we provide the details about data pre- and post processing for the remainder DES points.

Structural gadolinium-based T1 MR images were acquired either in Montpellier (306 patients) or Trento (50 patients), 3 months after surgery. The subject native T1 images were realigned with *fslreorient2std* (FSL v6.0.5.1)[7] to match the approximate orientation of the standard template image (MNI152 as provided by FSL, 1mm^3 isotropic spatial resolution), automatically skull stripped using *mri_synthstrip*[8] (Freesurfer v7.3.2), and linearly registered to the MNI template (1mm^3 isotropic spatial resolution, trilinear interpolation) using FSL flirt (flirt v6.0).[9,10] Due to the unavailability of the tumours' segmentations, an affine registration was used. Of note, affine transformations have been shown to provide a reliable alignment to the reference space specifically for glioma patients, in some cases resulting even more accurately than non linear methods.[1,11] Both skull stripping and registration outputs were visually inspected to detect gross abnormalities. In addition, for the newly acquired DES patients (starting N=356 patients, 8 patients removed due to incomplete brain coverage), we automatically segmented the brains in MNI space via Freesurfer's mri_synthseg command.[12] Reasoning that a fair comparison with the reference MNI brain would entail the selection of brain structures not heavily impacted by the tumour, we extracted the brainstem, the cerebellum, and the whole brain mask as regions of interest from both our set of 348 patients and from the MNI template (as provided by FSL[7]), and compared their overlap in MNI space via dice coefficient (scipy v1.7.3).[13] We found that a linear registration is robust enough, as illustrated by the robust median (min, max) dice coefficient values: 0.87 (0.72,0.95), 0.91 (0.77,0.95), and 0.96 (0.95,0.97) for cerebellum, brainstem, and brain mask, respectively (Supplementary Fig. 1).

Next, the stimulation coordinates were extracted from a csv file and written into a nifti file – 1 at the stimulated voxel, 0 otherwise – in the subject native space via *nibabel* [14] and *numpy*[15] (v3.2.2 and v1.20.3, respectively). Similarly to the preprocessing applied to the corresponding subject native T1, the nifti file was reoriented using *fslreorient2std* and linearly registered to the MNI template using the registration matrices obtained from the subject-to-template alignment. Finally, the coordinates (in mm) of the stimulated voxel in the MNI space were extracted with *fslstats* and stored in a csv file.

STEP: Realignment of cortical points to the gyri

After registration to the MNI space, cortical points were realigned to the gyri using the Destrieux atlas[16] as provided by FreeSurfer, obtained by running the non-skull stripped T1 image of the MNI template through FreeSurfer's *recon-all* routine. [17] From the Destrieux atlas, we extracted all the labels belonging to the gyri (with two additional labels per hemisphere not explicitly classified, i.e. "Pole occipital" and "Pole temporal"), shifting each cortical stimulation point toward the closest voxel – as per Euclidean distance – belonging to the aforementioned set of regions. Of note, the realignment was performed in the volumetric space to avoid too many data manipulation steps, as bringing single voxels to a surface representation of the brain is notoriously difficult and would have required moving the data back and forth across different spaces multiple times. Cortical stimulation points that had to be moved >= 5mm were discarded from the analysis. This step was undertaken to correct for possible errors arising from the manual annotation of the stimulation points, as they were by definition taken on the brain's gyral surface. 71 cortical points (corresponding to 2,3% of the starting dataset) were removed from the analysis, resulting in 3'000 valid cortical points distributed across 20 functional categories. Functional categories with less than 10 stimulations points or judged of no interest were discarded, resulting in a final selection of 2906 cortical and 12 functional categories**.**

STEP: Realignment of subcortical points to white matter and subcortical nuclei

Similarly to the cortical points, subcortical stimulation points were realigned to a brain mask encompassing the white matter and the deep subcortical nuclei and obtained by automatically segmenting the T1 image of the MNI template via FreeSurfer's *SynthSeg* tool.[12] Subcortical stimulation points that had to be moved >= 5mm were discarded from the analysis. 11 subcortical points (corresponding to 0,79% of the starting dataset) were removed from the study, resulting in 1'374 valid subcortical points distributed across 22 functional categories. We selected the stimulation coordinates of the 12 cortical categories, resulting in a final selection of 1231 subcortical stimulations.

From DES to functional networks

Our multimodal causal network mapping procedure aims at integrating DES with resting state fMRI (rsfMRI) via lesion network mapping (LNM).[18] By using the lesions location associated with a certain syndrome as seeds to derive group representative functional maps

obtained from a large cohort of healthy subjects, LNM is able to leverage the spatial heterogeneity in lesions location to extract a common neural substrate associated with that syndrome. Similarly, we treated each cortical stimulation point as a transient lesion, and used it as a seed for performing a seed based analysis (SBA)[19] in a normative connectome derived from 1'000 healthy individuals (GSP dataset).[20,21]

STEP: Seed based analysis (SBA) with the cortical stimulation points

For each tested function separately, cortical stimulation points were used as seeds for performing a SBA analysis[22,23] in the GSP dataset. For each subject and stimulation point, the average rsfMRI BOLD signal was extracted within a sphere of 5 mm radius – corresponding to spacing of the bipolar electrode used for stimulating the cortical surface – centred on the stimulation coordinates, and cross-correlated with every other grey matter voxel of the brain, mask computed with FreeSurfer's *SynthSeg* tool) to obtain a subject and DES points specific functional map. Before statistics, Pearson's *r* scores were transformed to *z* scores using Fisher's *r*-to-*z* transform via AFNI.[22] For each seed, representative group maps – one for positive correlations, and one for negative correlations – were obtained with a non-parametric one sample t-test (FSL's tool *randomise* with the Threshold-Free Cluster enhancement option enabled).[24] Due to the high number of both subjects and seeds, the number of permutations was reduced to 1'000. By providing connectivity maps at the voxel level, SBA allows a spatially unbiased mapping of whole brain connectivity, and combined with seeds derived from DES, it potentially allows to *causally* map the spatial topography of putative large scale functional networks subtending each tested functional category.

STEP: From seed maps to functional networks via lesion network mapping

Traditional LNM consists of three steps.[25] First, a group representative functional connectivity map – usually obtained with a parametric approach (e.g one sample t-test) – is computed for each seed. Subsequently, a high threshold (e.g. T-score) is applied to each image, resulting in a binary representation of functional connectivity. As a last step, binary images are aggregated, and a single frequency map is computed, with high concordance brain regions delineating the network of interest. The second step is where our approach and the traditional LNM differ the most, as we did not rely on an arbitrary and universal hard threshold for all DES-derived functional maps, but employed a non parametric procedure to threshold each DES connectivity map separately. Of note, lesion size represents a possible

confound in traditional LNM, a methodological artefact that is circumvented by using DES, as DES-derived seeds have equal volume.

Before aggregating the binary maps obtained from the non-parametric significance computation, we removed duplicated stimulation coordinates and possible outliers, the latter defined on the basis of the functional connectivity profile of all points within a given function. For each tested functional category separately, we computed the dice coefficient (scipy v1.7.3)[13] between all possible pairs of binary connectivity maps. A seed map was deemed as outlier if its minimum dice coefficient across all seeds for a given category was below 0.75. As for the realignment of the cortical points to the gyral surface, this step was undertaken to correct for possible errors arising from the manual annotation of the stimulation and to reduce the impact of isolated stimulations points. After the data cleaning procedure, additional 156 cortical stimulation points were removed. Of note, sensorial (16), motor (33) and speech arrest (62) points accounted for more than 70% of the removed points, with their removal mainly driven from the being repeated measurements in densely sampled functional categories. As a result, 2’750 cortical points were used for the LNM analysis.

STEP: Network thresholding

If not written otherwise, each grey matter voxel was assigned to either the positive or negative DES network according to the relative frequency values of the DES positive and DES negative networks (*winner takes it all principle*). Specifically, this principle applies for the whole brain networks representation of Fig. 2, and several additional analyses such as the comparison between DES derived networks and the probabilistic DES atlases, the robustness and specificity analyses, and the comparison between functional and structural connectivity.

Validation analyses

As reported in the main text, we implemented a series of analyses to probe the validity of our procedure (see *Validation* section).

STEP: resting state fMRI preprocessing

Acquisition parameters for the cohort of 66 patients were described elsewhere.[26] Briefly, structural and resting state fMRI (rsfMRI) images were acquired with a clinical 1.5 T GE Healthcare MRI Scanner at the Department of Radiology of Santa Chiara Hospital, Trento (Italy). For rsfMRI sessions (*total acquisition time*=12 min, *TR*=2.6s, 275 volumes), patients

were instructed to lie still, with eyes open, and to think of nothing. Structural scans included standard T1-weighted (T1w) anatomical and a T2/FLAIR for every patient. T1w images underwent bias field correction (N4 algorithm as provided by ANTs)[27,28], automatic skull stripping via *mri_synthstrip*, and automatic segmentation via *mri_synthseg*. Binary masks – eroded by one voxel via AFNI – were created for white matter, cerebrospinal fluid, and ventricles. Finally, T1w images were registered to the MNI template as provided by FSL (2mm^3 isotropic resolution). RsfMRI preprocessing was carried out with AFNI[22] and FSL[7], and consisted in removal of the first four volumes (*3dTcat*), despiking (*3dDespike*), slice timing correction (*3dTshift* with the wsinc9 option), motion correction (*mcflirt*), automatic skull stripping (*mri_synthstrip*), registration to the subject T1w image (*epi_reg*), nuisance regression (which included band pass filtering between 0.01 - 0.1 Hz, details below), registration to the MNI template (2mm^3 isotropic resolution), and smoothing with a full width at half maximum of 4mm (*3dBlurInmask*, with a mask encompassing cortical and subcortical grey matter. Registration was performed in one step by concatenating the epi-to-T1w and the T1w-to-MNI registration matrices with *convert_xfm* and applying *flirt* with a spline interpolation. Registration outputs were visually inspected to detect gross abnormalities. With respect to nuisance regression, we first flagged volumes contaminated by high motion, defined on the basis of a framewise displacement (FD) greater than 0.5mm (FD estimated with *fsl_motion_outliers*). Of note, uncensored segments of data containing fewer than five contiguous volumes were also flagged, similarly to Chen et al.[29] In a second step, we projected subject specific white matter and cerebrospinal fluid masks defined in the subject anatomical (i.e. T1w) space to the subject functional (i.e. EPI) space, extracting white matter and cerebrospinal fluid time series that were concatenated with the motion traces (six rotations and six translations) computed with *mcflirt*. For these eight regressors, we computed their temporal derivatives via AFNI's *1d_tool*, and used them as additional regressors. We used *3Tproject* to perform nuisance regression, which allowed us to automatically define the frequency bands of no interest (i.e. the ones above 0.1 and below 0.01 Hz) as additional regressors (*passband* option). In doing so, we were able to perform nuisance regression and bandpass filtering in one step, minimising data manipulation. Moreover, the volumes flagged with high motion were not removed from the analysis, but their values replaced by interpolated neighbouring (in time) non-flagged values before any projections (*ntrp* option). In doing so, the analysis proceeded without the actual removal of any time points.

STEP: Generalising from a clinical population to healthy subjects

We computed the degree of similarity (via Pearson correlation, corrected for spatial autocorrelation with BrainSMASH)[30] between the averaged network representations of the glioma patients with both DES and rsfMRI data available (*n*=34, see Supplementary Fig. 2 for lesion loci map) and the average network representations of the healthy population (*n*=1000), leveraging the DES points and functional imaging scans with preoperative functional imaging available. We restricted the comparison to functional categories of clinical interest – sensory-motor functions and language – and with at least 10 stimulation points available. Following these criteria, anomia (*n*=22), speech arrest (*n*=31), sensorial (*n*=12), and motor (*n*=30) were retained for further analysis. Single subject functional maps were created for each DES point, and averaged across the groups separately. To produce null maps with preserved spatial autocorrelation, the framework outlined in[30] and implemented in BrainSMASH employs a parametrized generative model centred on the estimation of the variance as a function of distance (a so-called variogram). At its core, the procedure consists in randomly permuting the values in a given brain map, reintroducing spatial autocorrelation via smoothing and re-scaling of the original, non permuted data. We used the default parameters as provided in BrainSMASH, creating 1000 surrogates for each of the 95 DES derived functional maps. Following the recommendations provided in [31] ,we also checked the goodness of the surrogate spatial maps by creating 100 additional surrogates with preserved autocorrelation properties that were compared – via Pearson correlation – with the corresponding original map. To reduce computational complexity, we employed state of the art cortical [32] and subcortical atlases,[33] as previous studies showed that parcellation has a negligible impact on the generation of the null maps.[31] As reported in the main text, the averaged network representation of the glioma patients did not differ from the average network representation of the healthy population for the subset of 95 DES points tested in the current report (average Pearson r = 0.85). Moreover, the goodness of fit analysis revealed that the default parameters were good enough to recapitulate the degree of spatial autocorrelation present in the empirical data. To probe this, for each of the 100 comparisons between the DES driven seed and its surrogates we extracted the median value of the distribution, averaging across DES-derived seeds to obtain a representative value. We found an average Person r value of 0.97, corroborating the use of default values for creating surrogates maps with a preserved spatial autocorrelation structure.

We repeated the analysis by comparing high grade glioma patients (n=20) and low grade glioma patients (N=14). We found that the averaged network representations of the two groups did not differ (average Pearson r=0.83). As for the comparison between the whole glioma sample and the healthy population, we found that the default parameters were good enough to recapitulate the degree of spatial autocorrelation present in the empirical data (average Pearson r values=0.96)

STEP: Specificity and robustness analyses

To further corroborate the predictive capacity of DES derived networks, we probed the specificity and robustness of our mapping procedure (Supplementary Fig. 3 and Supplementary Fig. 4, respectively). For a given functional category, specificity was tested by cross correlating – via Spearman correlation – the network frequency values obtained via cross validation (see Validation section in the main text) with the network frequency values of all other functional categories.

Robustness was assessed by estimating – for each functional domain – the functional network via lesion network mapping with a subset of the available direct electrical stimulation points (x axis, 1 to 90% percentage, 100 repetitions for each step), computing the overlap with the original empirical network via dice coefficient. For each subset, we reported the median dice score (y axis, black circles), 25th and 75th percentile (y axis, red errors bars).

The specificity analysis revealed category specific network signatures coexisting with the observation of largely overlapping functional systems, while the robustness analysis suggests that DES derived networks are highly resilient against random seed removal.

STEP: Repeated DES measurements of the same patient

To account for the possibility that heavily sampled patients may dominate the network building step, and hence dominating the spatial arrangement of the resulting functional networks, we re-computed the networks by retaining only one DES point per subject. This was done for the behavioural categories where the subject-unique set of DES points accounted for less than 75% of the total number of stimulations (n=8 functional categories). We computed similarity with respect to the full set of DES points using the eta^2 coefficient (0 no similarity, 1 exact similarity, as implemented in AFNI)[22,34]. Of note, eta^2 is stricter than correlational approaches, as it tests for exact similarity. Our analysis revealed that the DES

functional networks estimated with the reduced set of DES points are virtually indistinguishable from the DES functional networks estimated with the full set of stimulation points (eta^2 of 0.998, 0.999, 0.999, 0.996, 0.999, 0.999, 0.999, and 0.988 for anomia, motor, movement arrest, semantic, sensorial, speech arrest, verbal apraxia, and visual, respectively).

STEP: Probing the structural scaffold of DES functional networks

As mentioned in the main text, we used TractoInferno[35] (n=284) to test whether DES derived cortical networks are supported by the structural connectivity of the subcortical stimulations. For each functional category separately, we assigned each grey matter voxel to either the positive or negative DES network according to the relative frequency values of the two networks (*winner takes it all principle*). In doing so, we obtained an almost perfect bipartition of the cortical mantle, with every voxel assigned to one of the two networks. We then registered the T1 weighted image of each subject to the MNI template (as provided by FSL, 1mm^3 isotropic spatial resolution, trilinear interpolation) using FSL flirt (flirt v6.0, affine transformation). In a second step, subcortical DES points and DES-derived functional networks were projected back into the subject specific space by using the inverse registration matrix. For each subject, we computed the functional category specific grey matter/white matter interface by adding together the corresponding DES positive and DES negative networks mask obtained from the bipartition procedure, and feeding it as input for MRtrix's *5ttgen* command.[36] For each functional category separately, we also created a white matter mask to be used as a seeding mask for deterministic tractography, and derived from the intersection of the subject specific white matter mask and a mask obtained by drawing spheres of 5 mm radius at each subcortical stimulation site. We used deterministic tractography (SD_STREAM algorithm) as implemented in MRtrix, with the following parameters: *cutoff*=0.05, *step*=0.1, *angle*=30, *max_length*=250, *max_attempts_per_seed*=1000. One million of streamlines were generated for each functional category and each subject, and the -include and -stop options were used with respect to the functional category specific grey matter/white matter interface file. Following tractogram generation, we obtained a DES point specific tck file by filtering it with the MRtrix's tckedit command. Finally, we extracted the percentage of streamlines connecting each subcortical DES point to the corresponding DES positive and negative functional network with the *tckstats* command. To compute functional category specific statistics, we first averaged within subjects (i.e. across points), and then across subjects. For visualisation purposes, the

streamlines in Fig. 4 are visualised with reference to a recently published white matter atlas of the human brain.[37]

Single subject analysis

As reported in the main text, we implemented a multivariate analysis able to quantify the adherence of the single subject functional organisation to the mapping obtained at the group level (Fig. 5, main text). We initially restricted the computation to the same subset of 95 cortical DES points (see *Validation* section for more details) derived from the 34 patients with both DES and preoperative functional imaging available. For each point in this subset, we first computed single subject functional maps in the GSP dataset, sampling connectivity values for all other cortical DES points belonging to that functional category. This new dataset consisting of 1’000 examples and N-1 variables (i.e. cortical DES points belonging to a specific functional category) was then fed to a machine learning algorithm (Isolation Forests),[38,39] able to learn the boundaries of the multivariate distribution, and ready to classify new instances as either in- or outliers, with accompanying classification score (negative values for outliers, positive values for inliers). Before learning, principal component analysis was performed to retain the number of features explaining 80% of the variance, resulting in a number of features varying from 7 to 20 (median=15). For a single DES derived functional map, the algorithm was trained on the whole normative normative data (1’000 examples and a varying number of features depending on the specific functional map) and used to predict the scores of the clinical population. Internally to the Isolation Forest algorithm, each feature receives equal weight. Following the author’s recommendations [38], no hyper parameter optimization was performed, and thus we used the default parameters as per Scikit-learn implementation (100 estimators, no bootstrap). Importantly, given that we had no ground truth data to validate our choice, we let the algorithm estimate the proportion of outliers in the dataset, as outlined in the original work.[38] In a first validation step, we used the point specific classifier to test the 95 DES points belonging to the patients with preoperative functional imaging available. As reported in the main text, we then repeated the procedure to build a classifier specific for language and derived from stimulation points that fell – for each category independently – within hub territories of anomia, semantic, and phonological, including the functional scans of the glioma patients that underwent surgery without DES mapping (final *n*=66). The hub threshold was set at the 90th percentile of each functional

category as per cross validation procedure (*Validation* section), resulting in the selection of 102 hubs (76 for anomia, 17 for semantic, and 9 for phonological, respectively). Cross validation (leave one out) was also performed on the healthy population to build a reference distribution. As in the first analysis, principal component analysis was performed to retain the number of features explaining 80% of the variance (number of features varying from 7 to 10 (median=9). Hub points with a classification score above the median of the negative values of the reference population were labelled as strong outliers. Reasoning that comparing n=1'000 against n=66 would have represented a biased comparison, we employed a non parametric procedure to compare the two samples. Out of the 1'000 healthy control subjects, we randomly selected 66 participants, repeating the procedure 100'000 times (sampling with replacement). In each iteration, we performed a two sample t-test, comparing the randomly extracted 66 healthy subjects with the 66 patients. Focusing on the effect size (as per Cohen's d), our analysis revealed the two samples were characterised by Cohen's $d \leq 0.52$ for 80% of the times, thus indicating small/moderate differences in the functional architecture of the hub points (Supplementary Fig. 5).

Investigating psychiatric conditions with DES derived functional networks

Similarly to Joutsa et al.[21] , we checked for a spatial correspondence between the primary targets of transcranial magnetic stimulation devices cleared by the FDA for the treatment of major depressive[40] and obsessive compulsive disorders[41] and DES derived networks. We found an overlap in the dorsolateral prefrontal cortices, key regions of the DES derived mentalizing network. Of note, the left dorsolateral prefrontal cortex – the primary target of the FDA approved coils – was not present in the functional atlas built exclusively on DES points, as all cortical stimulations were in the right hemisphere (Supplementary Fig. 6, top row). Prompted by this observation, we then investigated possible alterations of the mentalizing network in autism spectrum disorder (ASD), a neurodevelopmental condition whose defining atypicalities in social behaviour are thought to reflect mentalizing deficits.[42] To highlight cross-methodological applications of our mapping, we correlated the fractional amplitude of low frequency fluctuations (fALLF, a proxy for the intensity of local spontaneous activity)[43,44] in the left and right dorsolateral prefrontal cortices of 125 ASD patients (ABIDE I preprocessed, see below for methodological details) with the corresponding social total subscore of the Autism Diagnostic Observation Schedule

(ADOS)[45], a standardised test for diagnosing autism. After data harmonisation and regression of sex, age, IQ, and in-scanner motion (mean framewise displacement), we found a moderate but statistically significant positive correlation (Spearman's *rho* = 0.21 and 0.22, for the left and right dorsolateral prefrontal cortices, respectively, *p values* < 0.02) between the intensity of local spontaneous activity in the dorsolateral prefrontal cortices and the level of impairment in social behaviour (i.e. higher social total subscore of the ADOS, Fig. 6). Intrigued by this observation, we also tested for global network alterations in a large sample of ASD patients and control subjects (ABIDE I preprocessed, 270 ASD patients and 289 controls), as a recent study showed that low frequency oscillatory power had a dramatic impact on normal network functioning in a physiologically accessible species.[23] In line with this hypothesis, after data harmonisation and regression of sex, age, IQ, and in-scanner motion (mean framewise displacement) we found that ASD patients were characterised by a moderate but pervasive and statistically significant decrease in global connectivity in both mentalizing network and at the whole brain level, as assessed with a multivariate procedure and using weighted degree centrality as a metric of interest (kernel two-samples test, *maximum mean discrepancy* = 0.006, *p value* < 0.02, 10'000 permutations for both test configurations, Supplementary Fig. 6, bottom row).

We derive the primary targets of transcranial magnetic stimulation devices cleared by the U.S. Food and Drug Administration for the treatment of major depressive and obsessive compulsive disorders from the corresponding accompanying publications,[40,41] and used to inspect the mentalizing network (Supplementary Fig. 6, first row). For the empirical analysis of this latter DES derived network, functional imaging and clinical data were downloaded from the Preprocessed Connectomes Project web site,[46] using data released by the Autism Brain Imaging Data Exchange (ABIDE) initiative. In accordance with HIPAA guidelines and 1000 Functional Connectomes Project/INDI protocols, all ABIDE datasets have been anonymized, with no protected health information included. We performed data selection according to the following criteria: 1) no missing age, sex, and IQ information; 2) high anatomical and functional imaging data quality, assessed as a positive review of three different raters; 3) percentage of volumes with a framewise displacement greater than 0.2 mm below or equal to 50%; 4) exclusion of collection sites providing less than five functional scans. Additionally, ADOS scores were included only if the diagnostic test was administered and scored by reliable research personnel. Following these data selection criteria, we included 289 control subjects and 270 ASD patients for the between group comparison, and 125 ASD

patients for the correlational analysis. We used the fractional amplitude of low-frequency fluctuations (fALFF) and weighted degree centrality data preprocessed with the Connectome Computation System pipeline, selecting the version with bandpass filtering (0.01-0.1 Hz) but no global signal regression. After data download, we aggregated the functional data at the regional level with the Brainnetome atlas, which consists of 246 cortical and subcortical regions.[47] For the correlational analysis, we extracted the regions corresponding to the right and left dorsal prefrontal cortices (areas A9/46d), while the whole set of regions and parcels overlapping with the mentalizing network were used for the between group comparison. We performed site harmonisation via ComBat as implemented in the NeuroHarmonize package, with sex, age, IQ, and in-scanner motion (mean framewise) displacement as additional covariate,[48] using the standardised residuals as input for the correlational analysis and the between group comparison. For this latter analysis, we used a kernel two sample test[49] with a radial basis function kernel (*gamma*=1.0/sigma^2, with sigma^2 defined as the squared median euclidean distance between the two groups) as implemented in Olivetti et al.[50] We computed significance via a non parametric permutation test (10.000 permutations). Of note, using two different parcellation schemes (Schaefer 400[51] and the 180 regions of Glasser et al.[32], both in combination with a subcortical parcellation[33]) did not affect the between group comparison result.

Supplementary References

1. Sarubbo S, Tate M, De Benedictis A, et al. Mapping critical cortical hubs and white matter pathways by direct electrical stimulation: an original functional atlas of the human brain. *NeuroImage*. 2020;205:116237. doi:10.1016/j.neuroimage.2019.116237
2. Ng S, Valdes PA, Moritz-Gasser S, Lemaitre AL, Duffau H, Herbet G. Intraoperative functional remapping unveils evolving patterns of cortical plasticity. *Brain*. 2023;146(7):3088-3100. doi:10.1093/brain/awad116
3. Sarubbo S, De Benedictis A, Merler S, et al. Towards a functional atlas of human white matter: Functional Atlas of White Matter. *Hum Brain Mapp*. 2015;36(8):3117-3136. doi:10.1002/hbm.22832
4. Tate MC, Herbet G, Moritz-Gasser S, Tate JE, Duffau H. Probabilistic map of critical functional regions of the human cerebral cortex: Broca's area revisited. *Brain*. 2014;137(10):2773-2782. doi:10.1093/brain/awu168
5. Roux A, Lemaitre AL, Deverdun J, Ng S, Duffau H, Herbet G. Combining Electrostimulation With Fiber Tracking to Stratify the Inferior Fronto-Occipital Fasciculus. *Front Neurosci*. 2021;15:683348. doi:10.3389/fnins.2021.683348
6. Sarubbo S, Tate M, De Benedictis A, et al. A normalized dataset of 1821 cortical and subcortical functional responses collected during direct electrical stimulation in patients undergoing awake brain surgery. *Data Brief*. 2020;28:104892. doi:10.1016/j.dib.2019.104892
7. Jenkinson M, Beckmann CF, Behrens TEJ, Woolrich MW, Smith SM. FSL. *NeuroImage*. 2012;62(2):782-790. doi:10.1016/j.neuroimage.2011.09.015
8. Hoopes A, Mora JS, Dalca AV, Fischl B, Hoffmann M. SynthStrip: skull-stripping for any brain image. *NeuroImage*. 2022;260:119474. doi:10.1016/j.neuroimage.2022.119474
9. Jenkinson M, Smith S. A global optimisation method for robust affine registration of brain images. *Med Image Anal*. 2001;5(2):143-156. doi:10.1016/S1361-8415(01)00036-6
10. Jenkinson M, Bannister P, Brady M, Smith S. Improved Optimization for the Robust and Accurate Linear Registration and Motion Correction of Brain Images. *NeuroImage*. 2002;17(2):825-841. doi:10.1006/nimg.2002.1132
11. Visser M, Petr J, Müller DMJ, et al. Accurate MR Image Registration to Anatomical Reference Space for Diffuse Glioma. *Front Neurosci*. 2020;14:585. doi:10.3389/fnins.2020.00585
12. Billot B, Magdamo C, Cheng Y, Arnold SE, Das S, Iglesias JE. Robust machine learning segmentation for large-scale analysis of heterogeneous clinical brain MRI datasets. *Proc Natl Acad Sci*. 2023;120(9):e2216399120. doi:10.1073/pnas.2216399120
13. Virtanen P, Gommers R, Oliphant TE, et al. SciPy 1.0: fundamental algorithms for scientific computing in Python. *Nat Methods*. 2020;17(3):261-272. doi:10.1038/s41592-019-0686-2
14. Brett, Matthew, Markiewicz, Christopher J., Hanke, Michael, et al. nipy/nibabel: 3.2.2. Published online June 6, 2022. doi:10.5281/ZENODO.6617121
15. Harris CR, Millman KJ, van der Walt SJ, et al. Array programming with NumPy. *Nature*. 2020;585(7825):357-362. doi:10.1038/s41586-020-2649-2
16. Destrieux C, Fischl B, Dale A, Halgren E. Automatic parcellation of human cortical gyri and sulci using standard anatomical nomenclature. *NeuroImage*. 2010;53(1):1-15. doi:10.1016/j.neuroimage.2010.06.010
17. Fischl B, Salat DH, Busa E, et al. Whole Brain Segmentation. *Neuron*. 2002;33(3):341-355. doi:10.1016/S0896-6273(02)00569-X
18. Fox MD. Mapping Symptoms to Brain Networks with the Human Connectome. *N Engl J Med*. 2018;379(23):2237-2245. doi:10.1056/NEJMra1706158
19. Lv H, Wang Z, Tong E, et al. Resting-State Functional MRI: Everything That Nonexperts Have Always Wanted to Know. *Am J Neuroradiol*. Published online January 18,

2018:ajnr;ajnr.A5527v1. doi:10.3174/ajnr.A5527
20. Buckner RL, Roffman JL, Smoller JW. Brain Genomics Superstruct Project (GSP). Published online 2014. doi:10.7910/DVN/25833
21. Joutsa J, Moussawi K, Siddiqi SH, et al. Brain lesions disrupting addiction map to a common human brain circuit. *Nat Med*. 2022;28(6):1249-1255. doi:10.1038/s41591-022-01834-y
22. Cox RW. AFNI: Software for Analysis and Visualization of Functional Magnetic Resonance Neuroimages. *Comput Biomed Res*. 1996;29(3):162-173. doi:10.1006/cbmr.1996.0014
23. Rocchi F, Canella C, Noei S, et al. Increased fMRI connectivity upon chemogenetic inhibition of the mouse prefrontal cortex. *Nat Commun*. 2022;13(1):1056. doi:10.1038/s41467-022-28591-3
24. Winkler AM, Ridgway GR, Webster MA, Smith SM, Nichols TE. Permutation inference for the general linear model. *NeuroImage*. 2014;92:381-397. doi:10.1016/j.neuroimage.2014.01.060
25. Boes AD, Prasad S, Liu H, et al. Network localization of neurological symptoms from focal brain lesions. *Brain*. 2015;138(10):3061-3075. doi:10.1093/brain/awv228
26. Saviola F, Zigiotto L, Novello L, et al. The role of the default mode network in longitudinal functional brain reorganization of brain gliomas. *Brain Struct Funct*. 2022;227(9):2923-2937. doi:10.1007/s00429-022-02490-1
27. Avants BB, Tustison NJ, Stauffer M, Song G, Wu B, Gee JC. The Insight ToolKit image registration framework. *Front Neuroinformatics*. 2014;8. doi:10.3389/fninf.2014.00044
28. Tustison NJ, Avants BB, Cook PA, et al. N4ITK: Improved N3 Bias Correction. *IEEE Trans Med Imaging*. 2010;29(6):1310-1320. doi:10.1109/TMI.2010.2046908
29. Chen J, Tam A, Kebets V, et al. Shared and unique brain network features predict cognitive, personality, and mental health scores in the ABCD study. *Nat Commun*. 2022;13(1):2217. doi:10.1038/s41467-022-29766-8
30. Burt JB, Helmer M, Shinn M, Anticevic A, Murray JD. Generative modeling of brain maps with spatial autocorrelation. *NeuroImage*. 2020;220:117038. doi:10.1016/j.neuroimage.2020.117038
31. Markello RD, Misic B. Comparing spatial null models for brain maps. *NeuroImage*. 2021;236:118052. doi:10.1016/j.neuroimage.2021.118052
32. Glasser MF, Coalson TS, Robinson EC, et al. A multi-modal parcellation of human cerebral cortex. *Nature*. 2016;536(7615):171-178. doi:10.1038/nature18933
33. Tian Y, Margulies DS, Breakspear M, Zalesky A. Topographic organization of the human subcortex unveiled with functional connectivity gradients. *Nat Neurosci*. 2020;23(11):1421-1432. doi:10.1038/s41593-020-00711-6
34. Cohen AL, Fair DA, Dosenbach NUF, et al. Defining functional areas in individual human brains using resting functional connectivity MRI. *NeuroImage*. 2008;41(1):45-57. doi:10.1016/j.neuroimage.2008.01.066
35. Poulin P, Theaud G, Rheault F, et al. TractoInferno - A large-scale, open-source, multi-site database for machine learning dMRI tractography. *Sci Data*. 2022;9(1):725. doi:10.1038/s41597-022-01833-1
36. Tournier JD, Smith R, Raffelt D, et al. MRtrix3: A fast, flexible and open software framework for medical image processing and visualisation. *NeuroImage*. 2019;202:116137. doi:10.1016/j.neuroimage.2019.116137
37. Radwan AM, Sunaert S, Schilling K, et al. An atlas of white matter anatomy, its variability, and reproducibility based on constrained spherical deconvolution of diffusion MRI. *NeuroImage*. 2022;254:119029. doi:10.1016/j.neuroimage.2022.119029
38. Liu FT, Ting KM, Zhou ZH. Isolation-Based Anomaly Detection. *ACM Trans Knowl Discov Data*. 2012;6(1):1-39. doi:10.1145/2133360.2133363
39. Pedregosa F, Varoquaux G, Gramfort A, et al. Scikit-learn: Machine Learning in Python. *J Mach Learn Res*. 2011;12(85):2825-2830.
40. Perera T, George MS, Grammer G, Janicak PG, Pascual-Leone A, Wirecki TS. The Clinical TMS Society Consensus Review and Treatment Recommendations for TMS

Therapy for Major Depressive Disorder. *Brain Stimulat*. 2016;9(3):336-346. doi:10.1016/j.brs.2016.03.010
41. Carmi L, Tendler A, Bystritsky A, et al. Efficacy and Safety of Deep Transcranial Magnetic Stimulation for Obsessive-Compulsive Disorder: A Prospective Multicenter Randomized Double-Blind Placebo-Controlled Trial. *Am J Psychiatry*. 2019;176(11):931-938. doi:10.1176/appi.ajp.2019.18101180
42. the EU-AIMS LEAP group, Moessnang C, Baumeister S, et al. Social brain activation during mentalizing in a large autism cohort: the Longitudinal European Autism Project. *Mol Autism*. 2020;11(1):17. doi:10.1186/s13229-020-0317-x
43. Mantini D, Perrucci MG, Del Gratta C, Romani GL, Corbetta M. Electrophysiological signatures of resting state networks in the human brain. *Proc Natl Acad Sci*. 2007;104(32):13170-13175. doi:10.1073/pnas.0700668104
44. Yu-Feng Z, Yong H, Chao-Zhe Z, et al. Altered baseline brain activity in children with ADHD revealed by resting-state functional MRI. *Brain Dev*. 2007;29(2):83-91. doi:10.1016/j.braindev.2006.07.002
45. Gotham K, Risi S, Pickles A, Lord C. The Autism Diagnostic Observation Schedule: Revised Algorithms for Improved Diagnostic Validity. *J Autism Dev Disord*. 2007;37(4):613-627. doi:10.1007/s10803-006-0280-1
46. Cameron C, Yassine B, Carlton C, et al. The Neuro Bureau Preprocessing Initiative: open sharing of preprocessed neuroimaging data and derivatives. *Front Neuroinformatics*. 2013;7. doi:10.3389/conf.fninf.2013.09.00041
47. Fan L, Li H, Zhuo J, et al. The Human Brainnetome Atlas: A New Brain Atlas Based on Connectional Architecture. *Cereb Cortex*. 2016;26(8):3508-3526. doi:10.1093/cercor/bhw157
48. Pomponio R, Erus G, Habes M, et al. Harmonization of large MRI datasets for the analysis of brain imaging patterns throughout the lifespan. *NeuroImage*. 2020;208:116450. doi:10.1016/j.neuroimage.2019.116450
49. Gretton A, Borgwardt KM, Rasch MJ, Schölkopf B, Smola A. A Kernel Two-Sample Test. *J Mach Learn Res*. 2012;13(null):723-773.
50. Olivetti E, Benozzo D, Kia SM, Ellero M, Hartmann T. The Kernel Two-Sample Test vs. Brain Decoding. In: *Proceedings of the 2013 International Workshop on Pattern Recognition in Neuroimaging*. PRNI ’13. IEEE Computer Society; 2013:128-131. doi:10.1109/PRNI.2013.41
51. Schaefer A, Kong R, Gordon EM, et al. Local-Global Parcellation of the Human Cerebral Cortex from Intrinsic Functional Connectivity MRI. *Cereb Cortex*. 2018;28(9):3095-3114. doi:10.1093/cercor/bhx179

Supplementary Table and Figures

Tumour Distribution	N
Frontal	173
Parietal	53
Temporal	109
Occipital	1
Insular	28
Para-Insular	139
Limbic/Prelimbic	79
Fronto-Parietal	15
Temporo-Parietal	7
Temporo-Occipital	8

Supplementary Table 1. Tumour distribution across major anatomical landmarks.

	N points (tot)	N points (right)	N points (left)	N unique patients	N Tum (right)	N tum (left)	N tum (bil)	Sex (M)	Sex (F)	N right hand	N left hand	N bil hand	Mean age	Std age	Male ratio	% Right hand
AMODAL ANOMIA	124	57	67	105	46	57	2	60	45	90	13	2	39.77	11.64	57.14	85.71
ANOMIA	304	16	288	196	13	183	0	108	88	178	12	6	39.31	10.73	55.1	90.82
MENTALIZING	137	127	10	90	80	8	2	47	43	77	10	3	39.84	12.58	52.22	85.56
MOTOR	795	434	361	381	178	200	3	216	165	326	42	13	39.3	11.5	56.69	85.56
MOVEMENT ARREST	535	307	228	303	144	156	3	163	140	264	32	7	38.63	11.17	53.8	87.13
PHONOLOGICAL	128	7	121	108	6	102	0	67	41	94	9	5	39.89	12.29	62.04	87.04
SEMANTIC	378	29	349	253	24	229	0	136	117	223	23	7	39.44	11.54	53.75	88.14
SENSORIAL	431	244	187	129	75	54	0	76	53	109	12	8	38.13	11.21	58.91	84.5
SPATIAL PERCEPTION	50	45	5	36	32	4	0	16	20	34	1	1	38.89	9.54	44.44	94.44
SPEECH ARREST	716	244	472	426	142	283	1	230	196	375	38	13	40	11.47	53.99	88.03
VERBAL APRAXIA	466	188	278	271	107	162	2	149	122	237	29	5	38.83	11.38	54.98	87.45
VISUAL	73	53	20	44	32	12	0	24	20	36	5	3	40.82	11.66	54.55	81.82

Supplementary Table 2. Summary statistics for the reported functional category, together for cortical and subcortical DES stimulations. DES stimulations on the sagittal midline (N=1, SENSORIAL, subcortical) were assigned to the right hemisphere.

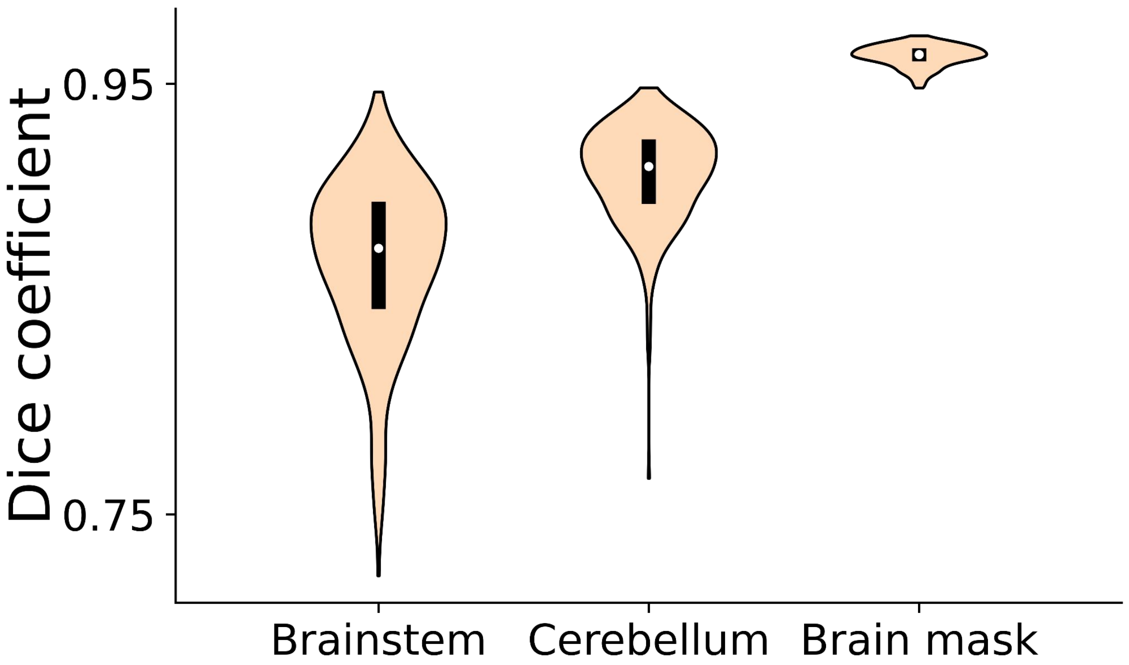

Supplementary Figure 1. Empirical Assessment of the goodness of the linear registration. For the newly acquired DES patients (starting N=356 patients, 8 patients removed due to incomplete brain coverage), we automatically segmented the brains in MNI space via Freesurfer's mri_synthseg command. Reasoning that a fair comparison with the reference MNI brain would entail the selection of brain structures not heavily impacted by the tumor, we extracted the brainstem, the cerebellum, and the whole brain mask as regions of interest from both our set of 348 patients and from the MNI template (as provided by FSL), and compared their overlap in MNI space via Dice coefficient. White dot: median. The lower and upper limits of the black bar represent the first and third quartile of the distribution, respectively.

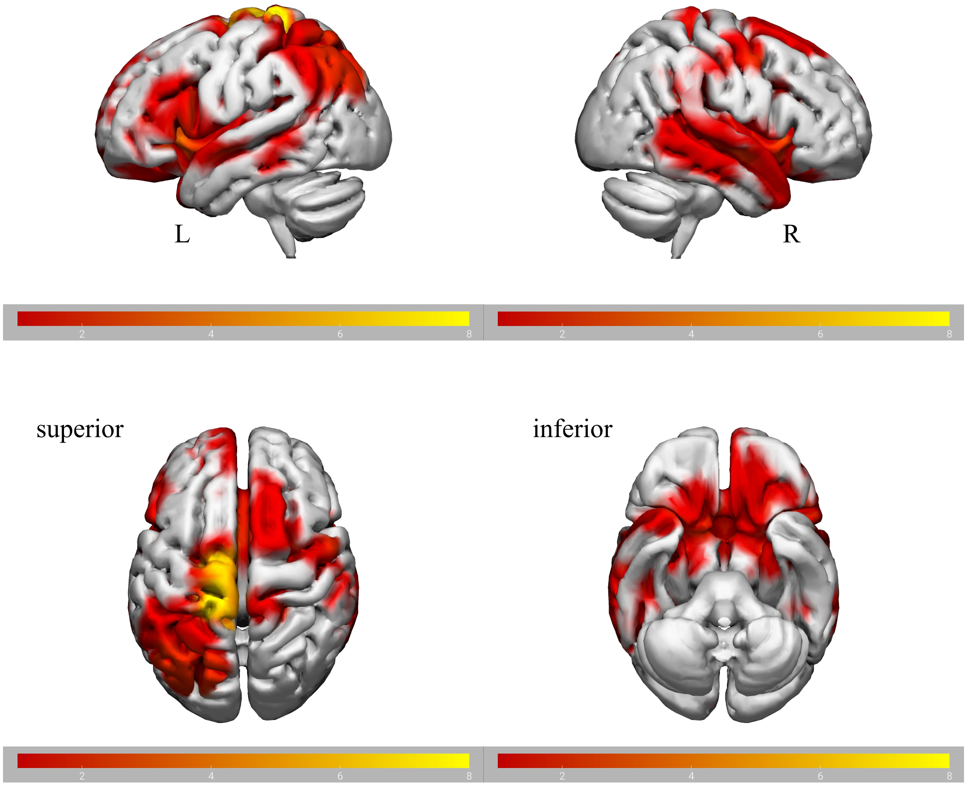

Supplementary Figure 2. Heat map showing tumor overlap across the 34 patients with both direct electrical stimulation and resting state fMRI data available. The heatmap was created by overlapping the tumours' cavities registered in MNI space.

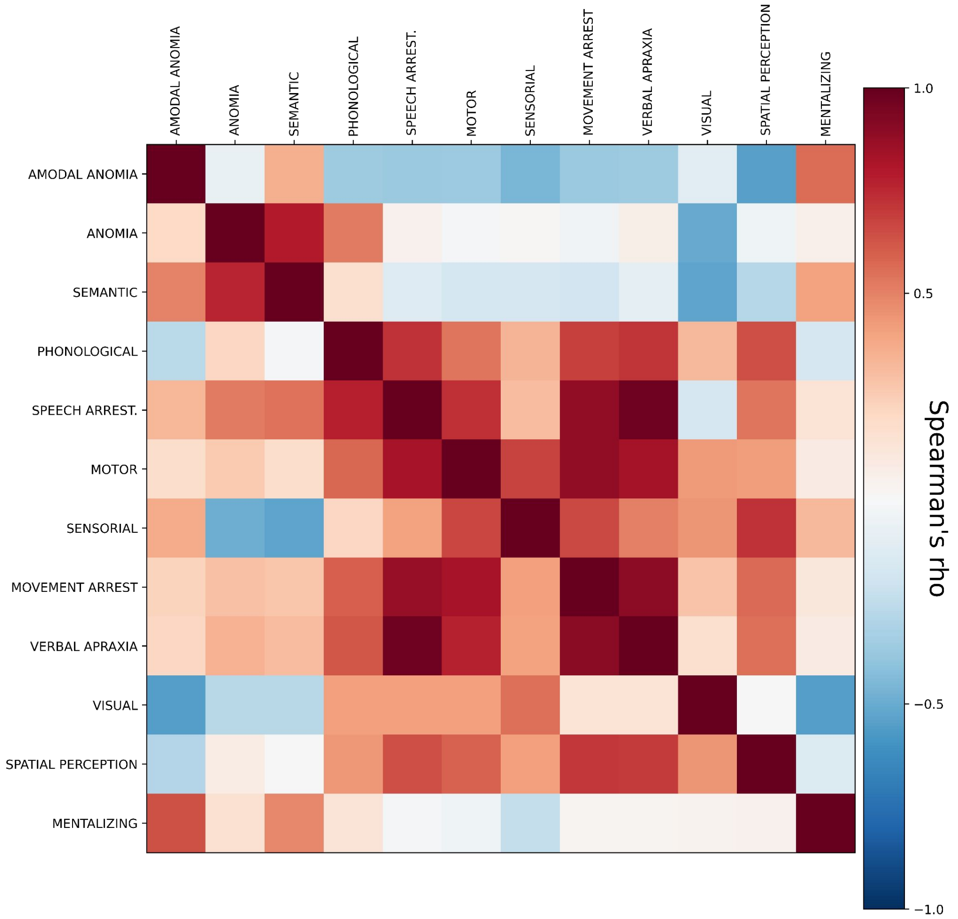

Supplementary Figure 3. Specificity analysis across functional categories. For each functional domain, we cross correlated – via Spearman correlation – its network frequency values obtained via cross validation (see *Validation* section in the main text) with the network frequency values of all other functional categories. The analysis revealed category specific network signatures – highest values always on the diagonal – coexisting with the observation of largely overlapping functional systems.

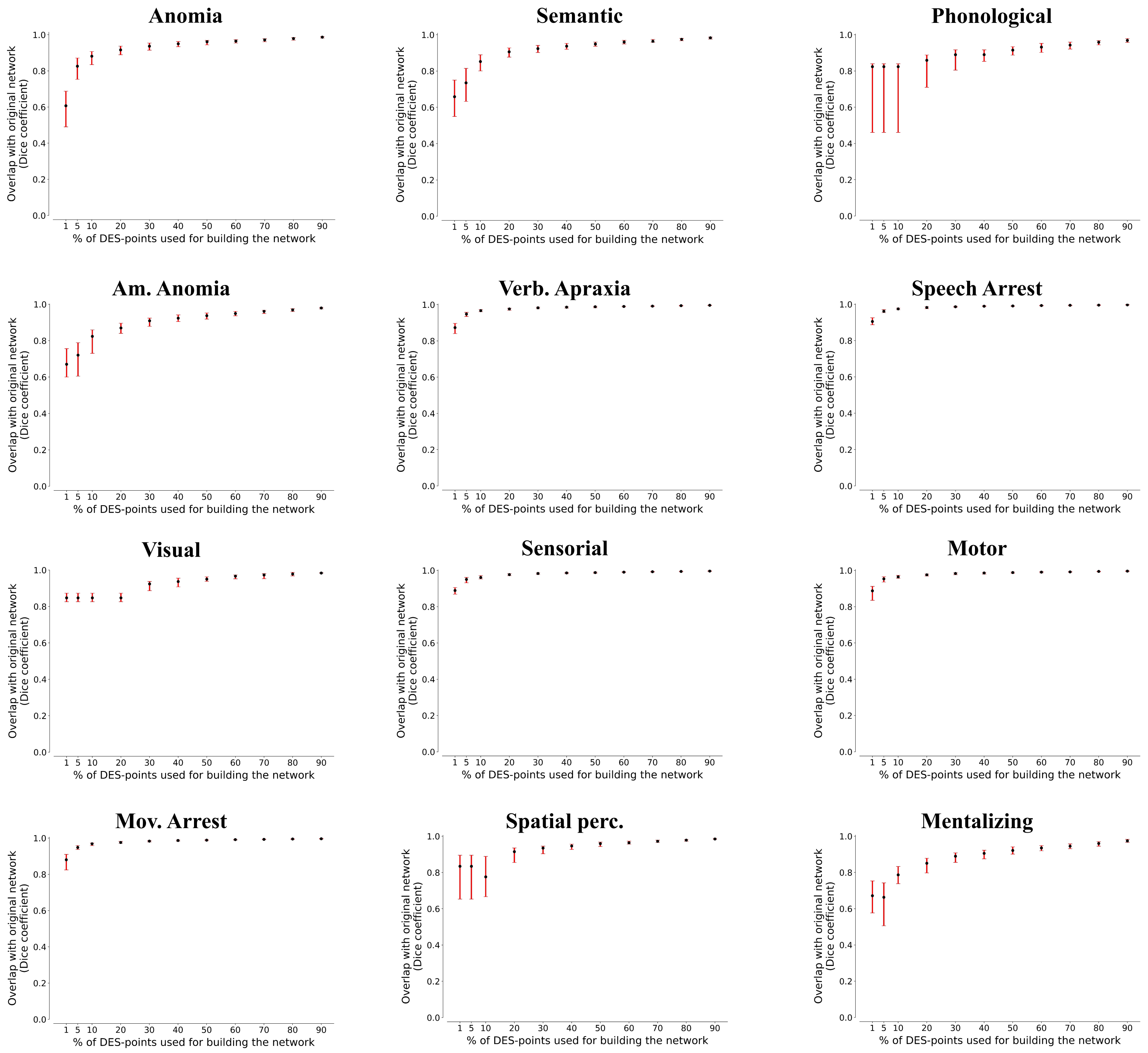

Supplementary Figure 4. Robustness analysis across functional categories. For each functional domain, we estimated the functional network via lesion network mapping using a subset of the available direct electrical stimulation points (x axis, 1 to 90% percentage, 100 repetitions for each step), computing the overlap with the original empirical network via dice coefficient. For each subset, we reported the median dice score (y axis, black circles), 25th and 75th percentile (y axis, red errors bars). Our results suggest that DES derived networks are robust against random seed removal.

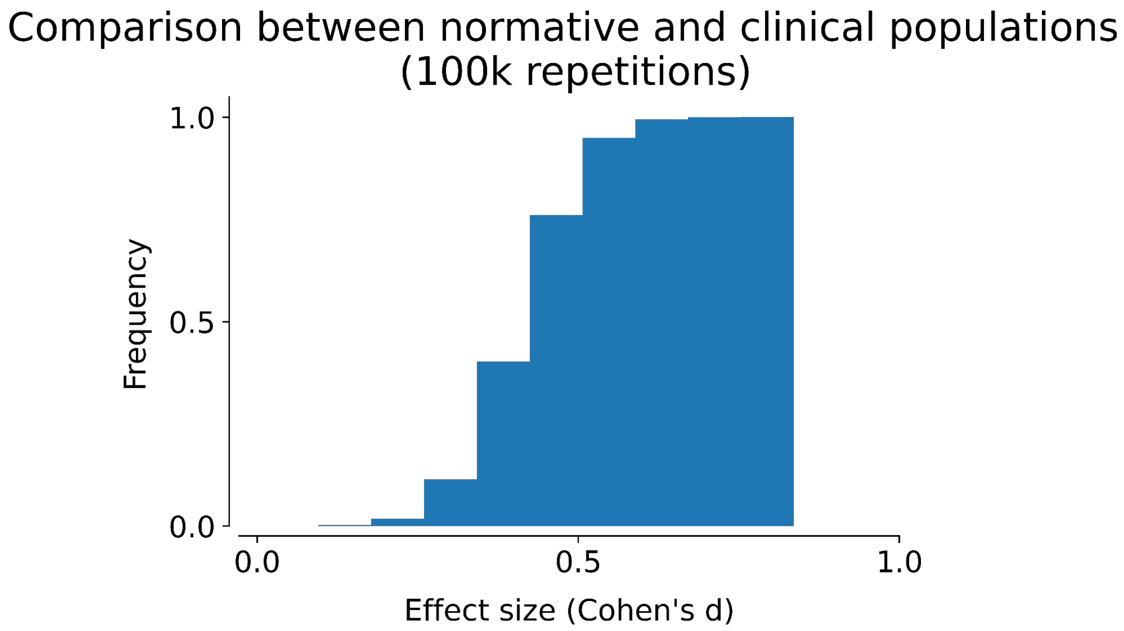

Supplementary Figure 5. Non parametric test to compare the distribution of outliers across the normative and clinical population. Out of the 1’000 healthy control subjects, we randomly selected 66 participants to match the sample size of the clinical population, repeating the procedure 100’000 times (sampling with replacement). In each iteration, we performed a two sample t-test to compare the percentage of hub points labeled as strong outliers across the two populations, extracting Cohen’s as a measure of interest. Our analysis revealed the two samples were characterized by Cohen's d <= 0.52 for 80% of the times, thus indicating small/moderate differences in the functional architecture of the hub points.

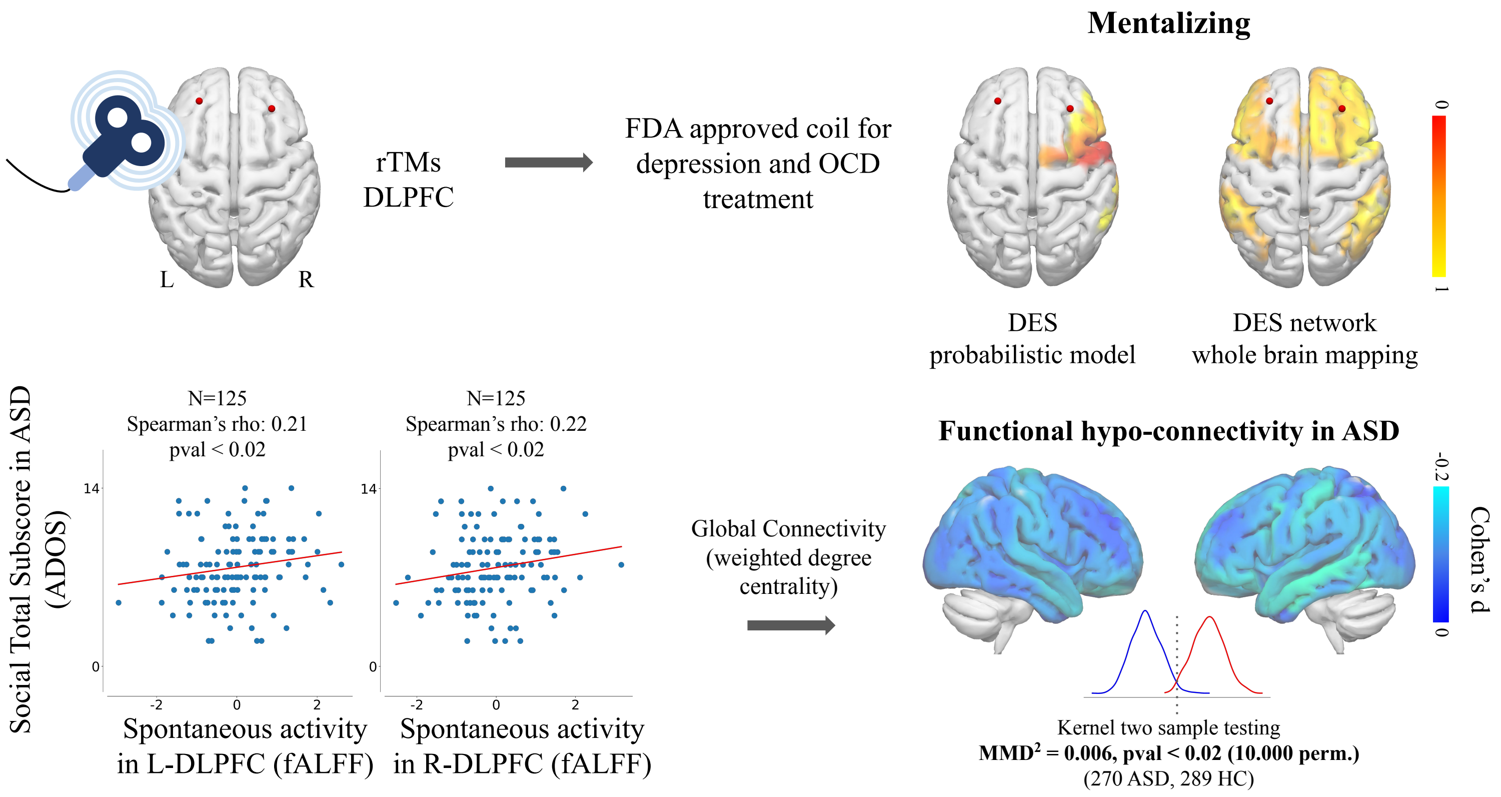

Supplementary Figure 6. Investigating psychiatric conditions with DES derived functional networks. The DES derived functional network of mentalizing overlaps with FDA approved transcranial magnetic stimulation coils for the treatment of major depressive disorder and obsessive compulsive disorder (upper row), and can be used to characterize brain-behavior relationship in neurodevelopmental conditions such as autism spectrum disorder (bottom row). Abbreviations: ADOS, Autism Diagnostic Observation Schedule; ASD, autism spectrum disorder; DES, direct electrical stimulation; DLPFC, dorsolateral prefrontal cortex; fALFF, fractional amplitude of low frequency fluctuations; FDA, Food and Drug administration; L, left; OCD, obsessive compulsive disorder; MMD, maximum mean discrepancy; R, right; rTMS, repetitive transcranial magnetic stimulation.
